# Dandelion-shaped strontium-gallium microparticles for the hierarchical stimulation and comprehensive regulation of wound healing

**DOI:** 10.1093/rb/rbae121

**Published:** 2024-10-18

**Authors:** Minrui Ji, Zaixin Yuan, Hongdong Ma, Xian Feng, Cong Ye, Lei Shi, Xiaodong Chen, Fei Han, Caichou Zhao

**Affiliations:** Department of Dermatology, Affiliated Hospital of Nantong University, Medical School of Nantong University, Nantong 226001, China; Department of Orthopaedics, Affiliated Hospital of Nantong University, Medical School of Nantong University, Nantong 226001, China; Department of Respiratory and Critical Care Medicine, Affiliated Hospital of Nantong University, Medical School of Nantong University, Nantong 226001, China; Department of Orthopaedics, Affiliated Hospital of Nantong University, Medical School of Nantong University, Nantong 226001, China; Department of Orthopaedics, Affiliated Hospital of Nantong University, Medical School of Nantong University, Nantong 226001, China; Department of Orthopaedics, Affiliated Hospital of Nantong University, Medical School of Nantong University, Nantong 226001, China; Department of Orthopaedics, Affiliated Hospital of Nantong University, Medical School of Nantong University, Nantong 226001, China; Department of Dermatology, Affiliated Hospital of Nantong University, Medical School of Nantong University, Nantong 226001, China; Department of Orthopaedics, Affiliated Hospital of Nantong University, Medical School of Nantong University, Nantong 226001, China; Department of Dermatology, Affiliated Hospital of Nantong University, Medical School of Nantong University, Nantong 226001, China

**Keywords:** hemostasis, wound healing, strontium-based microparticles, metal polyphenol network

## Abstract

The management of full-thickness skin injuries continues to pose significant challenges. Currently, there is a dearth of comprehensive dressings capable of integrating all stages of wound healing to spatiotemporally regulate biological processes following full-thickness skin injuries. In this study, we report the synthesis of a dandelion-shaped mesoporous strontium-gallium microparticle (GE@SrTPP) achieved through dopamine-mediated strontium ion biomineralization and self-assembly, followed by functionalization with gallium metal polyphenol networks. As a multifunctional wound dressing, GE@SrTPP can release bioactive ions in a spatiotemporal manner akin to dandelion seeds. During the early stages of wound healing, GE@SrTPP demonstrates rapid and effective hemostatic performance while also exhibiting antibacterial properties. In the inflammatory phase, GE@SrTPP promotes M2 polarization of macrophages, suppresses the expression of pro-inflammatory factors, and decreases oxidative stress in wounds. Subsequently, during the stages of proliferation and tissue remodeling, GE@SrTPP facilitates angiogenesis through the activation of the Hypoxia-inducible factor-1α/vascular endothelial growth factor (HIF-1α/VEGF) pathway. Analogous to the dispersion and rooting of dandelion seeds, the root-like new blood vessels supply essential nutrients for wound healing. Ultimately, in a rat chronic wound model, GE@SrTPP achieved successful full-thickness wound repair. In summary, these dandelion-shaped GE@SrTPP microparticles demonstrate comprehensive regulatory effects in managing full-thickness wounds, making them highly promising materials for clinical applications.

## Graphical abstract

**Figure:**
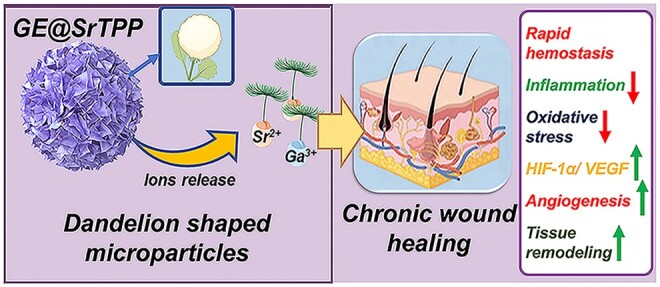

## Introduction

**Figure 1.**
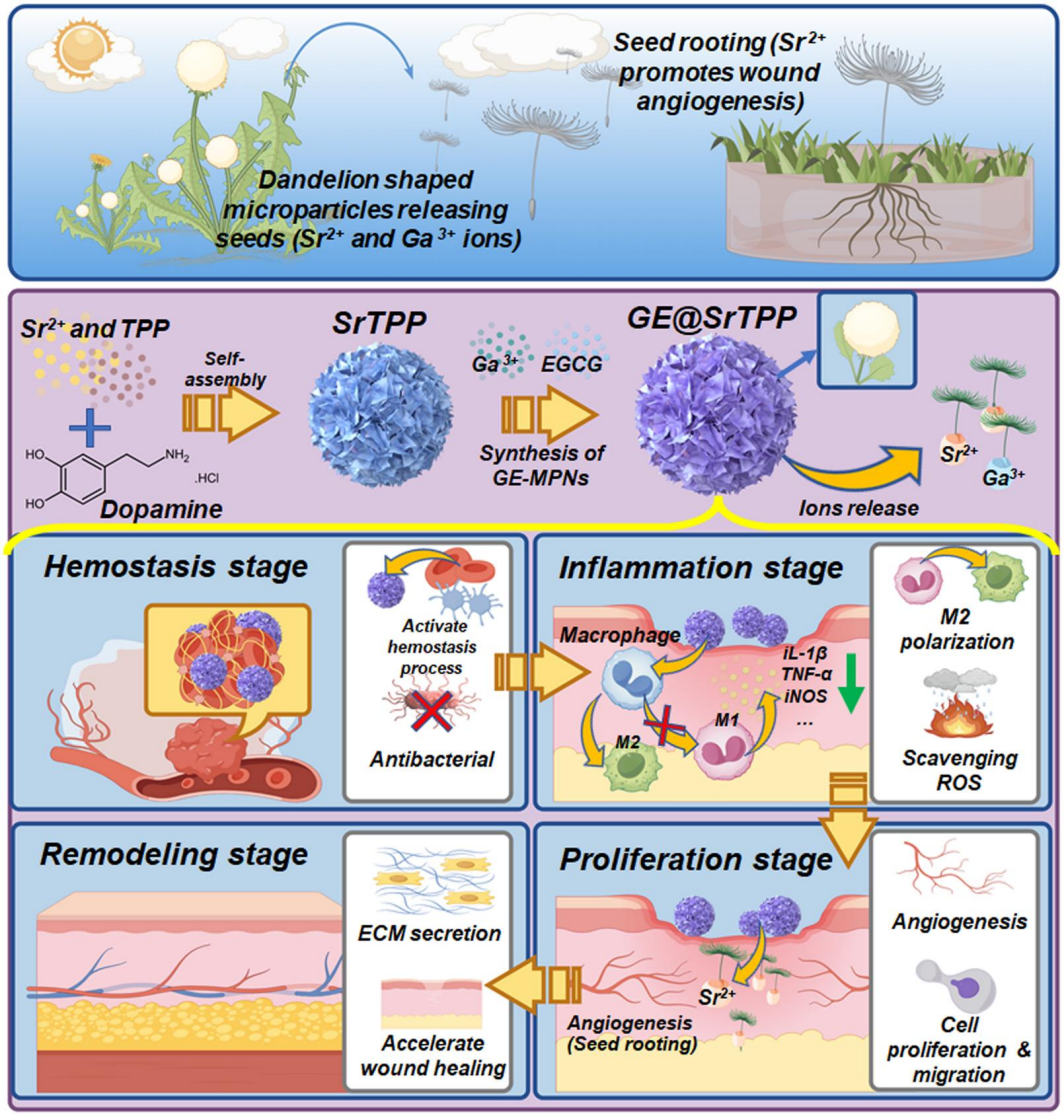
Schematic summarizing work on the preparation of dandelion-shaped strontium-gallium particles (GE@SrTPP) and their spatiotemporal regulation of biological processes in various stages of wound healing. GE@SrTPP are synthesized through dopamine-mediated Sr ion biomineralization and self-assembly, and then modified with Ga-MPNs. The morphology of GE@SrTPP resembles the mature inflorescence of dandelions, enabling the delivery of Sr and Ga ions akin to seeds. Consequently, GE@SrTPP can integrate functions such as rapid hemostasis, inflammation regulation, angiogenesis and skin tissue remodeling. Notably, during the proliferation stage, Sr ions can promote angiogenesis similar to dandelion seed rooting where the ‘root system’ (new blood vessels) provides nutrients for wound healing.

The process of skin wound healing is highly regulated and unfolds in a spatiotemporal manner, encompassing four distinct stages: hemostasis, inflammation, cell proliferation and tissue remodeling [1]. Hemostasis, as the initial biological response to injury, not only serves to prevent excessive blood loss and preserve life but also sets the stage for subsequent inflammatory and tissue healing processes [2–4]. However, traditional hemostatic methods involving the use of medical gauze for compression can lead to adhesion between new granulation tissue and the gauze during wound healing, resulting in pain, secondary injury and impeding subsequent tissue healing [5, 6]. During the inflammatory phase of wound healing, an excess of reactive oxygen species (ROS) and secreted pro-inflammatory factors in the wound microenvironment further contribute to prolonged inflammation and oxidative stress, which impedes normal healing or leads to scar formation [7, 8]. Previous studies have also demonstrated the efficacy of dressings with anti-inflammatory and ROS scavenging properties in accelerating wound healing, such as bioglass nanoparticles and nanoenzymes [9–12]. Furthermore, vascularization of regenerated skin tissue is a crucial indicator of successful wound healing due to disrupted local blood circulation post-injury [13]. While various hemostasis/wound care dressings have been proposed over recent decades with some success in accelerating wound healing—including gelatin sponge, montmorillonite QuikClot powder and HemCon dressing [14–16], most strategies focus on enhancing specific stages of healing rather than providing a comprehensive approach for spatiotemporal regulation and overall enhancement throughout all phases of wound repair [17, 18]. Hence, it remains a formidable task to propose a spatiotemporal regulatory system capable of achieving prompt hemostasis, inflammation modulation and skin tissue remodeling [18]. This integrated approach is designed to address the diverse requirements of various stages in the wound healing process as well as more intricate clinical demands (Figure 1).

In recent years, there has been increasing attention on the application of inorganic materials for emergency hemostasis and wound repair. Common inorganic hemostatic materials, such as zeolite, montmorillonite and kaolin, have been utilized for wound hemostasis due to their rapid plasma absorption, blood cell concentration and clot formation capability [19–21]. Nevertheless, these materials exhibit drawbacks such as thermal release, cellular toxicity and inadequate hemostatic efficacy, thereby constraining their clinical utility [19–21]. In recent years, numerous novel functional wound dressings have been proposed, and the incorporation of calcium-based inorganic materials into dressings has emerged as a prevalent research strategy. For instance, Zheng *et al.* [22] developed an aerogel doped with ultra-long calcium phosphate nanowires that efficiently achieved hemostasis and accelerated wound regeneration. Yu *et al.* [23] proposed a sea cucumber-inspired aerogel doped with hydroxyapatite nanoparticles for rapid hemostasis after bone trauma. Strontium (Sr) shares similar chemical properties with calcium and can also activate platelets while exhibiting additional biological activities such as promoting vascularization, antibacterial effects and inflammatory regulation [24–26]; however, there have been few reports on Sr-based hemostatic powder. In recent years, gallium (Ga) ions have garnered significant attention for their applications in bone regeneration, antibacterial properties and hemostasis [27]. Ga ion competes with Fe ion to bind ferritin in bacterial cytoplasm leading to metabolic imbalance and bacterial death due to its chemical similarity with Fe ion [28]. Additionally Ga^3+^ demonstrates ability in the intrinsic coagulation pathway by activating coagulation factors [27]. In conclusion, the combination of Ga and Sr may be a potential candidate for preparing synthetic spatiotemporal modulators at various stages of skin wound healing. The advantage of the metal system prepared by strontium and gallium, in comparison to the metal system prepared by Bochani *et al.* [29, 30] and Alinezhad *et al.* [31], is that it does not require light and heat irradiation, making it simpler to use.

Previous studies have demonstrated that positively charged molecules, such as dopamine hydrochloride, can trigger the self-assembly and biomineralization of calcium ions and triphosphates to produce mesoporous and uniform microparticles [32, 33]. This mesoporous structure can absorb body fluid, thereby accelerating the aggregation and adhesion of red blood cells (RBCs) and initiating the hemostatic process [34–36]. Drawing inspiration from this principle, we synthesized dandelion-shaped Sr-based microparticles (SrTPP) and further functionalized them with epigallocatechin gallate (EGCG)-Ga metal polyphenol networks (MPNs) (GE@SrTPP). EGCG is a representative plant polyphenol with catechol structures capable of coordinating with metal ions under neutral or alkaline conditions to form a MPNs [37, 38]. We hypothesize that these Sr–Ga composite dandelion-shaped microparticles can modulate different stages of wound healing through the sequential release of Ga^3+^ and Sr^2+^. In the early stage of healing, Ga^3+^ and EGCG confer rapid hemostatic function to the microparticles while exerting antibacterial, anti-inflammatory and ROS scavenging effects to eliminate negative factors during early-stage healing. In the intermediate to advanced stages of the healing process, Sr^2+^ released by microparticle plays a crucial role in regulating inflammation, facilitating angiogenesis and expediting wound closure. The sequential release of strontium and gallium ions resembles dandelion seeds ‘drifting’ in wounds; particularly noteworthy is how strontium ions ‘take root’ in wounds by promoting angiogenesis akin to a complex root system that delivers oxygen and nutrients essential for tissue regeneration. Overall, our study investigates the physicochemical properties of GE@SrTPP while conducting *in vitro* experiments to elucidate its coagulation mechanism and biological activity. Additionally, we evaluate its hemostatic efficacy using liver puncture bleeding models as well as tail-amputation bleeding models along with assessing its potential for repairing full-thickness chronic skin defects in diabetic rat models.

## Materials and methods

### Materials

The reagents required for material synthesis, including anhydrous strontium chloride, gallium nitrate, EGCG, trisodium phosphate, dopamine hydrochloride and acetic acid were purchased from Aladdin (Shanghai, China). Catalase assay kit and the hydroxyl radical scavenging capacity assay kit were from Macklin. The reagents required for cell culture, including Dulbecco’s modified Eagle medium (DMEM), fetal bovine serum and sterile phosphate-buffered saline (PBS), were purchased from Gibco (USA). Streptozocin (STZ, 98%), sodium citrate, lipopolysaccharide (LPS) and citric acid monohydrate were provided by Yuanye Biotech (Shanghai, China). The cells required for this study, human umbilical vein endothelial cells (HUVECs), fibroblasts (L929) and macrophages (Raw264.7), were purchased from Oricell^®^ (ScienCell Research Laboratories, Guangzhou, China).

### Synthesis of GE@SrTPP

The synthesis of dandelion-shaped microspheres (GE@SrTPP) was first carried out according to a modified report [32]. In brief, dopamine hydrochloride was dissolved in 1 wt% acetic acid solution at a concentration of 2 mg/ml to form a precursor solution. A 100 mg/ml tripolyphosphate sodium solution was prepared with deionized water. 1.2 ml of the tripolyphosphate sodium solution were gradually added to 3 ml of the precursor solution while stirring at 60°C in a water bath for 10 minutes. Then, 5 ml of 100 mM strontium chloride solution were added to the mixture and stirred at 60°C for another 2 hours. After the reaction was complete, the precipitate was collected by centrifugation at 5000 rpm, washed with deionized water twice, and then lyophilized to obtain strontium-based dandelion-shaped microspheres, which were named SrTPP. And then, on this basis, E@SrTPP and GE@SrTPP were synthesized. In brief, EGCG was dissolved in Tris solution (1 mM, pH8.0) at a concentration of 1 mg/ml. Different concentrations of gallium(III) nitrate were added to the EGCG-Tris solution to obtain precursor solutions of MPNs with different gallium concentrations (gallium concentrations of 0.1, 0.2 and 0.5 mg/ml, respectively). The obtained precursor solutions of MPNs were centrifuged at 12 000 rpm and the precipitates were discarded, and the supernatants were retained. Fifty milligrams of SrTPP was added separately to 40 ml solutions of different gallium ion concentrations of MPNs precursors, and the pH of the solution was adjusted to 8.0 with 1M NaOH solution by slow stirring for 24 hours. After centrifuging at 5000 rpm and washing with deionized water twice, the resulting GE@SrTPP particles were lyophilized and stored. Based on the gallium ion concentration (0.1, 0.2 and 0.5 mg/ml), the obtained GE@SrTPP particles were named GE@SrTPP1, GE@SrTPP2 and GE@SrTPP3, respectively. The particles that were treated with the precursor solution without gallium were named E@SrTPP (i.e. only containing EGCG, without gallium element).

### Characterization of GE@SrTPP

The microscopic morphology of SrTPP, E@SrTPP, and each group of GE@SrTPP particles was observed using a transmission electron microscope (TEM, H-8000, Hitachi) and analyzed based on TEM equipped with an energy dispersive X-ray spectrometer (EDS) to determine the elemental composition of each group of particles. Additionally, the chemical structure of each group of samples was tested using Fourier transform infrared spectroscopy (FT-IR, TENSOR 27, Bruker) and X-ray diffractometer (XRD, D8 Advance, Bruker, Germany). The yields of E@SrTPP and GE@SrTPP are obtained by calculating the quality. The size distribution and average particle size of each group of particles were measured and statistically analyzed using Image J software based on TEM images. The materials of each group after ultrasound were observed under TEM to evaluate the stability of their structures. The extracts of GE@SrTPP was prepared by adding the materials into normal saline at a solid/liquid ratio of 40 mg/ml, and then incubated at 37°C for 1, 3, 7 and 14 days. The concentration of strontium ions and gallium ions in the collected solution was measured using an inductively coupled plasma optical emission spectrometer (ICP). Equal mass of each group material was added to normal saline, and the degradation rate of each group was calculated. The stability of GE@SrTPP at different time points was analyzed by TEM. Additionally, the free radical scavenging activity of GE@SrTPP was evaluated using the 1,1-diphenyl-2-picrylhydrazyl (DPPH·, Maclin, China) method. In brief, SrTPP, E@SrTPP, and each group of GE@SrTPP particles were dispersed at a concentration of 100 μg/ml in 2 ml of DPPH· solution (0.1 mM) and incubated for 30 minutes. Then, the supernatant was collected by centrifugation, and the absorbance at 517 nm was measured using a UV–vis spectrophotometer. Additionally, GE@SrTPP with different mass ratios (25, 50 and 100 mg/ml) was dispersed in DPPH solution and fully mixed and incubated for 30 minutes, and the absorbance at 517 nm was measured using UV–vis. The catalase assay kit and hydroxyl radical scavenging scavenging assay kit were used to detect the scavenging ability of GE@SrTPP to H_2_O_2_ and ·OH. The microplate reader was used to quantify the absorbance, and the scavenging efficiency was calculated [39]. In relation to the release of EGCG and dopamine, the procedural steps were consistent with those previously outlined, and supernatants were collected at various time points. The concentration of dopamine in the solution was quantified using a dopamine assay kit (Sangon, Shanghai, China) following the manufacturer’s protocol. Additionally, absorbance measurements at 273 nm were obtained using a visible-ultraviolet spectrophotometer to assess the total phenolic concentration; subsequently, the concentration of dopamine was subtracted to determine the concentration of EGCG.

### Evaluation of *in vitro* hemostasis efficiency

SD rats were obtained from the Experimental Animal Center of Nantong University, and the animal experiments were conducted in accordance with the approved protocol (S20240405-006) of the Experimental Animal Ethics Committee of Nantong University. All surgical procedures and postoperative care protocols strictly adhered to the guidelines for the use and care of animals. Briefly, fresh whole blood was collected from SD rats and immediately placed in tubes containing heparin sodium for a series of *in vitro* hemolysis and hemostasis studies. The blood clotting index (BCI) test was performed according to a modified procedure as previously reported [23]. In brief, 100 μl of fresh blood was dropped onto 100 mg particles; then the supernatant was collected at different time points after a 1 ml PBS wash, and its absorbance at 540 nm was measured. For the hemolysis experiment. The samples (10 mg) were co-cultured with 500 μl of 10% (v/v) blood cell suspension at 37°C for 1 hour. Subsequently, centrifugation was performed at 1000 rpm for 10 minutes, and the supernatant from each group was collected to measure absorbance at 540 nm. Deionized water and PBS were employed as negative and positive controls respectively. The hemolysis rate was calculated using Eq. (1):

(1) $$\mathrm{Hemolysis}\;\mathrm{rate}=\frac{\left(Bs-Bp\right)}{\left(Bw-Bp\right)}\times 100\%\;$$

where *Bs* represents the absorbance at 540 nm of the sample suspension, *Bp* denotes that of PBS and *Bw* corresponds to deionized water.

### Blood cell adhesion

Blood cell adhesion was assessed by incubating 100 mg of each sample in a preheated 24-well plate at 37°C for 10 minutes, followed by the addition of 1 ml of fresh citrate-anticoagulated rat blood to each well and further incubation at 37°C for 1 hour. Platelet adhesion was evaluated by adding platelet-rich plasma to the samples in the wells, also followed by an incubation at 37°C for 1 hour. Subsequently, the samples were washed with PBS three times to remove unadhered blood cells and platelets from the surface. The fixed samples were then dehydrated with an ethanol gradient. After dried, all samples were observed using scanning electron microscopy (SEM). Additionally, deionized water was added to separate RBCs and platelets in each group. The absorbance at 540 nm was recorded for the supernatant. RBC adhesion was quantified using the Eq. (2)

(2) $$\mathrm{RBC}\;\mathrm{adhesion}=\frac{As}{Ab}\times 100\%$$

where *As* is the absorbance of the blood serum in contact with the sample at 540 nm, and *Ab* is the absorbance of the control group at 540 nm.

Platelet adhesion was quantified using the Eq. (3)

(3) $$\mathrm{Platelet}\;\mathrm{adhesion}=\frac{As}{Ab}\times 100\%$$

### *In vivo* hemostasis test

The animal experiments were conducted in accordance with the approved procedures by the Experimental Animal Ethics Committee of Nantong University Experimental Animal Center (S20240405-006). All surgical procedures and postoperative care plans were strictly implemented following the guidelines for the use and care of animals. The hemostasis ability of the microparticle was assessed using a liver puncture bleeding model and rat tail-amputation bleeding model. The rats were divided into five groups: (i) gauze group, (ii) gelatin sponge group, (iii) SrTPP group, (iv) E@SrTPP group and (v) GE@SrTPP. Prior to the experiment, all samples were disinfected with ultraviolet irradiation. Anesthesia was induced with 3–5% isoflurane gas through an inhalation anesthesia machine, followed by maintenance under anesthesia with 1.5–3% isoflurane. After successful anesthesia, surgical preparation involved exposing and cleaning the liver before creating a deep wound by puncturing it five times with a 1.28 mm needle, allowing free bleeding for 20 seconds, and then applying each sample on the bleeding site until hemostasis occurred. Hemostasis time was measured using a stopwatch timing, and a precision quartz balance was employed for weighing. Additionally, for the rat tail-amputation model, the tail was cut off by surgical scissors in anesthesia, causing bleeding, and then exposed to the air for 15 seconds to allow for natural hemorrhage. The microparticles were evenly applied to the wound surface for hemostasis, and the bleeding time and mass of the post-bleeding sample were recorded.

### *In vitro* antibacterial properties of GE@SrTPP

The antibacterial activity of the microparticles was assessed using plate counting on agar with *Staphylococcus aureus* (*S. aureus*, ATCC25923) and *Escherichia coli* (*E. coil*, ATCC25922) as the experimental subjects. Briefly, a bacterial suspension (2 × 10^6^ CFU ml^-1^) was incubated with an equal amount of sterilized sample in a shaking incubator at 37°C and 120 rpm for 24 hours. The control group consisted of the suspension without the sample, and each solution was diluted by 1000 times. Subsequently, 100 μl of the evenly distributed solution were plated on Lysogeny broth (LB)- agar plates, and after 24 hours, the number of colonies on these plates was recorded. Simultaneously, a 1000 rpm centrifugation was conducted on a portion of the remaining bacterial suspension to collect the residual bacteria. The bacteria were then subjected to three washes with 0.85% NaCl solution through centrifugation, followed by resuspension in a bacterial live-dead staining solution and incubation in darkness for 15 minutes. Subsequently, 5 μl of the bacterial suspension were dispensed onto a coverslip, covered with another coverslip, and observed under an inverted microscope. The sediment from the remaining bacterial suspension was also subjected to centrifugation to collect the precipitate, which was then washed thrice with PBS before being fixed in a 2.5% glutaraldehyde solution. This was followed by sequential dehydration and drying using ethanol gradients before observation under SEM. The survival area of the bacteria was calculated using the Eq. (4)

(4) $$\mathrm{Survival}\;\mathrm{area}\%=\frac{St}{S0}\times 100\%$$

where *St* represents the area after co-cultivation with the sample and *S*_0_ is the area of untreated bacteria on agar plates.

### Biocompatibility of GE@SrTPP

Biocompatibility was assessed according to the international standard (ISO/EN 10993-5) for the preparation of particle extraction solution. Briefly, a mixture of microparticle and DMEM was prepared at a ratio of 200 mg/ml, shaken in a 37°C shaker at 120 rpm for 24 hours, followed by centrifugation and filtration with a 0.22 micron bacterial filter. The obtained extraction solution was then diluted to concentrations of 1/4, 1/8, 1/16, 1/32, 1/64 and 1/128. HUCVECs cells and L929 cells were seeded at a density of 5000 cells per well on a 96-well plate and treated with different concentrations of GE@SrTPP leaching solution for evaluation of cell proliferation using CCK8 assay after culture for both one and three days. Cell viability detection was performed using live/dead staining solution (Dojindo, Japan) on the first and third days with a concentration of the extraction solution at dilution factor of 1/16.

### Scratch wound-healing assay

The effect of hemostatic powder on cell migration *in vitro* was evaluated by scratch test. HUVECs were seeded at a density of 3 × 10^5^ cells per well in six-well plates and cultured for 24 hours. After the cells had fully adhered, a straight scratch was made on the cell surface using the tip of a 200-μl pipette and was gently washed twice with PBS. The cells were then cultured with the extraction solution for 24 hours and stained with live/dead dye for imaging by fluorescence microscope (Zeiss, Germany). The scratch assay was conducted on L929 cells in a similar manner. The migration images were observed and quantified using ImageJ, with the repair ratio calculated using the Eq. (5)

(5) $$\mathrm{Repair}\;\mathrm{ratio}\%=\left(1-\frac{S1}{S0}\right)\times 100\%$$

where *S*_1_ and *S*_0_ represent the wound area on day 0 and 24 hours, respectively.

### Tube formation *in vitro*

In brief, for tubule formation, a 24-well plate was placed on ice and 200 μl of matrix collagen gel (Corning, USA) was added to each well. HUVECs cells were then seeded into the wells and cultured with the 1/16 extraction solution of each sample. The tubules were observed 8 hours after cell seeding. Additionally, the cells were labeled with cell cytoskeleton fluorescent stain (Alexa Fluor™, ThermoFisher, USA) and the vessel capillary length and branching were analyzed using ImageJ. Furthermore, the expression levels of angiogenesis-related proteins (eNOS, HIF-1a, VEGF) were verified by western blot (WB), with HUVECs seeded at a density of 3 × 10^5^ cells per well in six-well plates and cultured for 24 hours. All primary antibodies were purchased from Proteintech (Wuhan, China). The Raw data for WB are detailed in Supplementary Figure S22.

### Reactive oxygen species scavenging ability of GE@SrTPP

The ability of each group of microparticles to clear ROS in cells was assessed using the ROS fluorescent probe DCFH-DA (Dojindo, Japan). HUVECs were seeded at a density of 1.0 × 10^5^ cells per well in 24-well plates. The cells were treated with 200 μM H_2_O_2_ in serum-free medium for 12 hours, followed by replacement of the medium with a 1/16 concentration of the extract solution. After 24 hours, the ROS level in the cells was measured using DCFH-DA staining, and the stained cells were analyzed using fluorescence microscopy and flow cytometry.

### Anti-inflammatory potential of GE@SrTPP

The study investigated the anti-inflammatory potential of different groups of scaffolds using Raw264.7 cells. Briefly, Raw264.7 cells were seeded at a density of 3 × 10^4^ per well in 24-well plates. After 24 hours, all cell groups were stimulated with LPS (10 ng/ml) for 12 hours to induce polarization toward the pro-inflammatory M1 phenotype. Following stimulation, the complete culture medium containing the extract (1/16 dilution ratio), and Raw264.7 cells were cultured for 3 and 7 days as described previously. The expression levels of genes and proteins related to cell polarization in Raw264.7 were assessed using quantitative polymerase chain reaction (Q-PCR) and immunofluorescence staining as mentioned earlier. Genes associated with M1 polarization and pro-inflammatory factors included C-C Chemokine Receptor Type 7 (CCR7), Inducible Nitric Oxide Synthase (iNOS), interleukin (IL)-1β and tumor necrosis factor (TNF)-α, while genes linked to M2 polarization and anti-inflammatory factors comprised Arg-1, CD206, IL-10 and IL-1ra. The extraction, purification, reverse transcription and Reverse Transcription-Polymerase Chain Reaction (RT-PCR) of mRNA were performed according to the instructions of the reagent manufacturer (Trizol, Reverse transcription kit, and RT-PCR kit were all purchased from Vazyme, Nanjing, China). The primer sequences involved in this study are detailed in Supplementary Table S1. RT-PCR was performed using the QuantStudio Q7 real-time system (Appliedbiosystems, USA). Gene expression relative to the housekeeping gene (GAPDH) was assessed by the comparative Ct (2^−^^ΔΔCT^) method. For immunofluorescence staining, Raw264.7 cells were cultured for 3 days in complete medium containing extraction solution and then fixed with 4% paraformaldehyde solution. Then, Arg-1 and iNOS (Proteintech, Wuhan, China) was labeled with fluorescence for analysis of the role of microparticles in inducing cell M1/M2 polarization. In addition, the cell cytoskeleton and nucleus were separately labeled with CellMask (Alexa Fluor™, ThermoFisher, USA) and DAPI dye (Dojindo, Japan), respectively.

### *In vivo* antimicrobial properties of GE@SrTPP

To validate the antibacterial properties of GE@SrTPP *in vivo*, we established a bacterial-infected skin wound model [40]. The animal experiments were conducted in accordance with the approved procedures by the Experimental Animal Ethics Committee of Nantong University Experimental Animal Center (S20240722-001). Before surgery, rats were adaptively fed for 1 week. During the experimental procedure, anesthesia was induced using isoflurane inhalation prior to creating four full-thickness wounds (10-mm diameter each) on the dorsal region of the rats. 50 μl *S. aureus* (10^8^ CFU/ml) was injected into the wound to induce wound infection. One day later, each group of materials was filled in the wound separately. To prevent scratching or chewing, a 3M film was pasted around the wound. Images of the wounds in each group were captured on days 1, 3, 7, 14 and 16 after treatment and the changes in wound size over time were analyzed by ImageJ. The percentage change in wound area from day *n* = 1/3/7/14/16 relative to day *n *= 0 was calculated as (At/A0)*100%. *S. aureus* was isolated from the wound and cultured on LB solid medium to observe bacterial growth and assess the antimicrobial efficacy of each group. On days 3, 7 and 16, rats were euthanized by inhalation of carbon dioxide and skin samples were collected from the wound modeling area for histological analysis. Specimens were fixed in 10% neutral formalin and then sectioned in paraffin. Wound healing was evaluated by H&E, Masson, TNF-α, and IL1-β staining. The biocompatibility of GE@SrTPP was evaluated by H&E staining of heart, liver, spleen, lung and kidney.

### *In vivo* wound repair experiments

In this work, the wound-healing effects of GE@SrTPP on full-thickness wounds in diabetic rats were examined using an *in vivo* model. Briefly, SD rats were given a single injection of freshly dissolved STZ (45 mg/kg) in a 0.1 M citrate buffer (pH 4.5) into peritoneum. One week later, the rats with fasting random blood glucose levels greater than 16.7 mmol/L were selected as the type I diabetic rats for experiments. During the experimental procedure, anesthesia was induced using isoflurane inhalation prior to creating four full-thickness wounds (8-mm diameter each) on the dorsal region of the rats and applying different formulations of hemostatic powder evenly onto each wound site. To prevent scratching or chewing, a 3M film was pasted around the wound. Post-surgery, individual housing was provided for the rats and wound-healing progress was monitored on days 1, 3, 7, 9, 12 and 14. The percentage change in wound area from day *n* = 1/3/7/9/12/14 relative to day *n* = 0 was calculated as (At/A0)×100%. For histological analysis of diabetic rat wounds at day 14, the animals were euthanized via carbon dioxide inhalation and skin samples from the wound modeling region collected. The overall observation of neovascularization at the wound site in various treatment groups was also documented [41]. Following fixation in 10%neutral formalin, the specimens were paraffin-embedded for sectioning. Histological examination included H&E, Masson, TNF-α, IL-1β, CD86, CD206, CD31 and Sirius red staining to assess wound healing. Immunofluorescence staining targeting proteins such as Alpha Smooth Muscle Actin (α-SMA), HIF-1α and iNOS, were performed to analyze inflammation, new vessel formation, and collagen synthesis. In addition, the hearts, livers, spleens, lungs and kidneys of the rats were stained with H&E to evaluate the biocompatibility of GE@SrTPP.

### Statistical analysis

Statistical analysis was performed using IBM SPSS Statistics 22 software. The data were reported as the mean accompanied by the standard deviation. To assess potential statistically significant differences among independent groups, analysis of variance was employed for multiple group comparisons, and post hoc Least Significant Difference (LSD) tests were conducted to evaluate statistical distinctions.

## Results

### Characterization of GE@SrTPP

**Figure 2.**
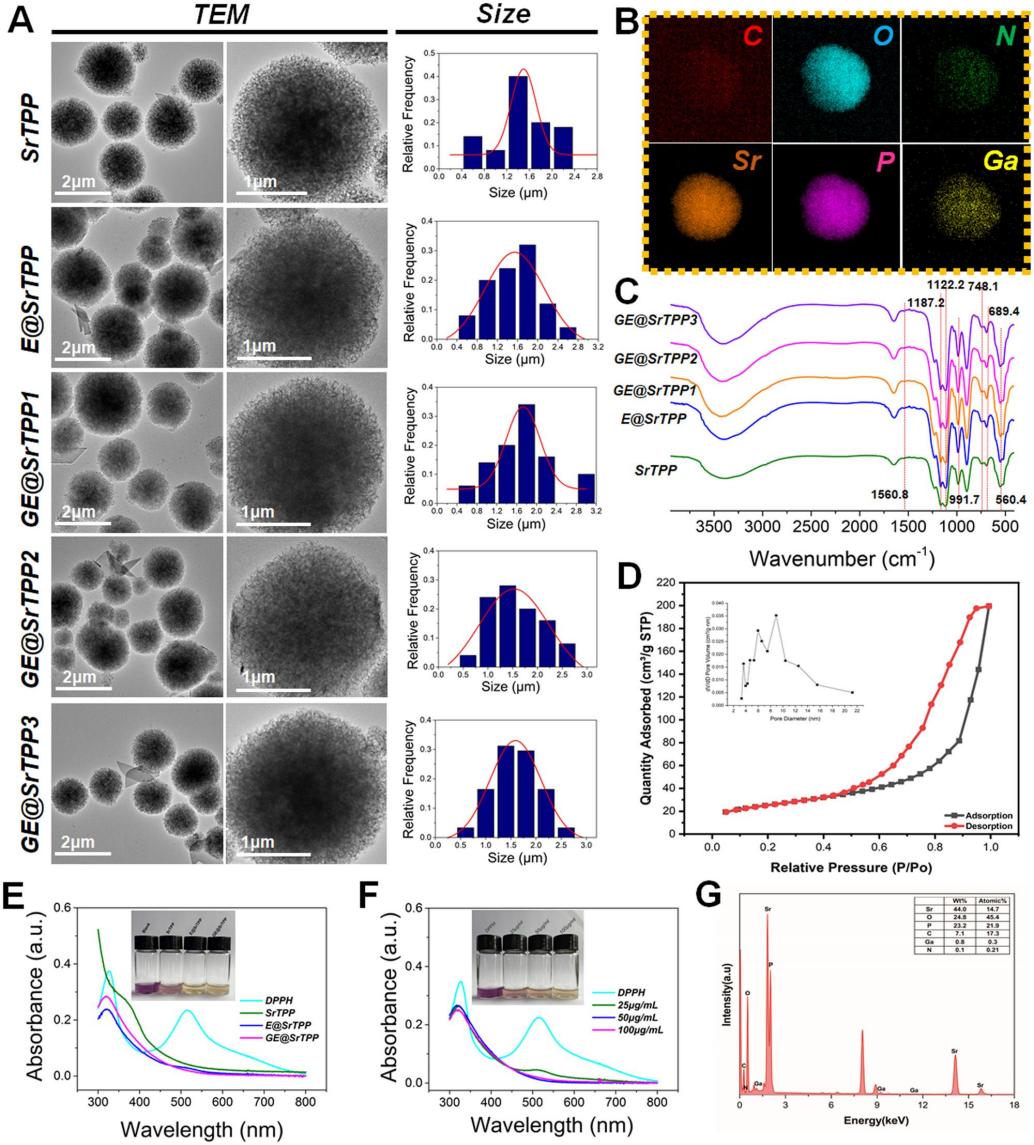
Characterization of dandelion-like microparticles (GE@SrTPP). (**A**) Transmission electron microscopy (TEM) images showed the morphology and size distribution of each group of GE@SrTPP. (**B**) Mapping analysis of GE@SrTPP2, displaying the composition of various elements in the microparticles. (**C**) FT-IR analysis of each group of dandelion-like microparticles. (**D**) N_2_ absorption–desorption isotherms and pore size distribution of GE@SrTPP2. (**E**) UV–vis spectra and photographs (inset) of DPPH after culture with different materials for 30 minutes. (**F**) UV–vis spectra of DPPH inhibition triggered by different concentrations of GE@SrTPP for 30 minutes. (**G**) EDS analysis reveals the composition and relative proportions of the elements present in GE@SrTPP.

The SrTPP was synthesized through the induction of dopamine, based on Sr ions and trisphosphate self-assembly [32]. Furthermore, it was modified with Ga-containing MPNs to obtain GE@SrTPP, as depicted in Supplementary Figure S1. During the preparation process, different concentrations of Ga^3+^ (0.1, 0.2 and 0.5 mg/ml) were added to the MPNs precursor fluid to prepare microparticles with different Ga contents. The resulting products were named GE@SrTPP1, GE@SrTPP2 and GE@SrTPP3; and the only EGCG-modified SrTPP was named E@SrTPP. The yields of E@SrTPP and GE@SrTPP were 93% and 87%, respectively. Firstly, the microscopic morphology of each group of particles was observed using TEM, as shown in Figure 2A. SrTPP, E@SrTPP and each group of GE@SrTPP exhibited a morphology similar to that of mature dandelion inflorescence—consisting of a large number of 2D nanoplatelets self-assembled which resulted in mesoporous morphology for the particles. The modification with MPNs did not have any significant effect on particle morphology or size distribution (Supplementary Figure S2); indicating that it did not alter the microscopic structure of SrTPP. There was a significant disparity in the elemental composition of the particles within each group, as illustrated in Figures 2B and Supplementary Figure S3. The mapping analysis results revealed the presence of gallium in GE@SrTPP2, with similar deposition observed in GE@SrTPP1 and GE@SrTPP3. Conversely, the SrTPP and E@SrTPP particle groups did not exhibit any gallium elements. This observation was further confirmed by line scan energy spectrum analysis (Supplementary Figure S4), which indicated a gradual increase in gallium distribution from the edge to the center of the particles, suggesting uniform dispersion rather than surface-only localization. Moreover, an increase in gallium ion concentration within the MPNs preparation system corresponded to higher gallium content within prepared particles (from GE@SrTPP1 to GE@SrTPP3). These findings collectively support successful preparation of MPNs containing gallium-modified SrTPP.

Additionally, data of Fourier infrared spectroscopy (FT-IR) revealed that absorption peaks at 748.1 and 689.4 cm^−1^ corresponding to symmetric bridge stretching of P–O bonds, along with peaks at 1122.2 and 1187.2 cm^−1^ related to P–O stretching vibrations; adsorption at 991.7 cm^−1^ due to symmetric terminal P–O stretching vibrations; and absorption at 560.4 cm^−1^ attributed to phosphate group rocking vibrations [42, 43]. Based on FT-IR spectra and previous reports, SrTPP was identified as a mixture of Sr_2_P_2_O_7_ and Sr_3_(PO_4_)_2_ [44–46]. Furthermore, the E@SrTPP to GE@SrTPP3 groups exhibited weak absorption peaks at 1560.8 cm^−1^, which were attributed to the extension–contraction vibrations of the C=C bond in EGCG. Additionally, the absorption peaks from 3200 to 3500 cm^−1^ were intensified due to the abundant presence of phenolic hydroxyl groups in EGCG [47]. Brunel–Emmett–Teller (BET) measurements were performed to evaluate the porosity of GE@SrTPP2 nanostructures. As depicted in Figure 2D, GE@SrTPP2 exhibit typical type IV N2 absorption–desorption isotherms, which are one of the main features of microporous materials. The BET surface area of GE@SrTPP2 was 87.7372 m^2^/g. The pore size distribution results (Figure 2D) shown that the pore width of GE@SrTPP was mainly concentrated in nanopores, and the average pore diameter was 9.01 nm. In the context of hemostatic powder, the advantage of this mesoporous structure lies in its ability to facilitate rapid absorption of bodily fluids, enhance hematocrit levels, promote blood cell aggregation and initiate the coagulation process [34]. The XRD pattern of the SrTPP sample reveals a broad scattering at lower angles, indicating the presence of long-range structural disorder and the amorphous nature of SrTPP. Furthermore, as shown in Supplementary Figure S5, the XRD pattern closely resembles that of previously reported strontium phosphate glass, suggesting that SrTPP primarily consists of amorphous Sr_3_(PO_4_)_2_ [46]. There were no discernible differences in the XRD patterns between groups that SrTPP modified with EGCG or MPNs. In comparison to SrTPP, diffraction peaks from EGCG appeared at 18.35° in each group [47]. Previous studies have demonstrated that the XRD pattern of polydopamine (PDA) exhibits a broad amorphous peak at 23.2° [48], lacking any crystalline reflections. However, as illustrated in Supplementary Figure S5, the XRD patterns of all groups do not display the characteristic peaks associated with PDA, suggesting that dopamine is present in its monomeric form when combined with strontium ions and triphosphate ions. EDS (surface scanning) results also revealed the presence of Sr, O, P, C, and a trace amount of Ga (Figure 2G). In conclusion, this study successfully synthesized dandelion-shaped Sr–Ga nanoparticles with a porous structure, primarily comprised of amorphous Sr_2_P_2_O_7_, Sr_3_(PO_4_)_2_ and Ga-EGCG MPNs.

Due to the ability of Ga ions to mimic the function of Fe ions, they have the potential to interfere with cellular processes related to Fe metabolism [27]. Therefore, in this study, we initially evaluated the biocompatibility and hemostatic properties of GE@SrTPP1, GE@SrTPP2 and GE@SrTPP3 in order to identify the most suitable Ga content for wound repair. As depicted in Supplementary Figure S6, HUVECs and fibroblasts (L929) cells cultured with different diluted extracts for 24 hours exhibited low toxicity and enhanced cell proliferation when exposed to GE@SrTPP1 and GE@SrTPP2 extracts. Conversely, GE@SrTPP3 demonstrated a significant inhibitory effect on cell proliferation. In the initial BCI experiment (Supplementary Figure S7), both the GE@SrTPP2 group and GE@SrTPP3 group displayed lower BCI values, highlighting the crucial role of Ga doping in enhancing early hemostasis function of SrTPP in skin injury. Therefore, in the subsequent studies, we selected GE@SrTPP2 as the experimental group for further research and renamed it GE@SrTPP. Subsequently, we validated the antioxidant properties of GE@SrTPP using the 2,2-diphenyl-1-picrylhydrazyl (DPPH·) method. DPPH is a stable purple free radical compound with a characteristic peak at 517 nm in its UV–vis spectrum. As depicted in Figure 2E, digital photographs demonstrate that the purple color of the DPPH· solution diminishes within 30 minutes when mixed with SrTPP, E@SrTPP and GE@SrTPP. Notably, the purple hue of the DPPH· solution completely vanishes in both E@SrTPP and GE@SrTPP groups, indicating a free radical scavenging rate exceeding 90%. The UV–vis spectrum revealed a significant decrease of the DPPH· characteristic peak at 517 nm in the SrTPP, E@SrTPP and GE@SrTPP groups, indicating their antioxidant properties. The SrTPP exhibited certain antioxidant properties due to its dopamine content, while the antioxidant properties of the particles were further enhanced after modification by EGCG or MPNs. Moreover, the free radical scavenging behavior was found to be concentration-dependent, with GE@SrTPP being able to rapidly eliminate DPPH· within 30 minutes at a concentration of 100 μg/ml (Figure 2F). At the same time, E@SrTPPh and GE@SrTPP also showed strong ability to scavenge H_2_O_2_ and ·OH (Supplementary Figure S8). These results validate the free radical scavenging activity of dandelion-shaped particles, which is dependent on the antioxidant mechanism of polyphenol. Overall, dandelion-shaped particles demonstrate excellent antioxidant activity and have potential for alleviating excessive oxidative stress in acute wounds and playing an anti-inflammatory role during wound healing. Subsequently, the *in vitro* ion release behavior of GE@SrTPP was confirmed by ICP analysis. The majority of Ga^3+^ were released within the first three days, with continued release for up to 7 days, indicating their potential antibacterial and hemostatic effects during the early stages of wound healing. Additionally, a sustained release of Sr^2+^ over 14 days was observed, suggesting their capability of regulating the intermediate to advanced stage of wound healing via the long-term release of Sr^2+^ (Supplementary Table S2). These findings indicate that GE@SrTPP exhibits potential spatiotemporal regulation functions for wound healing. We further investigated the stability and degradation characteristics of the particles. As illustrated in Supplementary Figure S9, the SrTPP, E@SrTPP, GE@SrTPP particles retained their dandelion-like morphology following ultrasonic treatment. After a 14-day degradation period. The degradation rates of E@SrTPP and GE@SrTPP were higher than that of SrTPP (Supplementary Figure S10). TEM images of the residual material indicated that the morphology of GE@SrTPP particles was progressively compromised over this duration, rather than undergoing complete degradation within a short timeframe (Supplementary Figure S11). The ICP data (Supplementary Table S2) and EDS analysis (Supplementary Figure S12) revealed that a majority of Ga^3+^ were released within 7 days, resulting in minimal Ga content in the residue. In contrast, Sr^2+^ were released in limited quantities during the initial phase but sustained long-term ion release, with substantial amounts remaining in the residue. Consequently, Ga^3+^ and Sr^2+^ exhibit distinct spatial and temporal release profiles that synergistically contribute to their effects at various stages of wound healing.

### The hemostatic efficiency and biocompatibility of GE@SrTPP

Biocompatibility is a fundamental requirement for wound dressings. Previous data have identified the optimal Ga-MPNs component-modified SrTPP, and it was found that a 1/16 concentration of the extract solution significantly enhanced cell proliferation. Consequently, the biocompatibility of all particle groups was further assessed. Supplementary Figure S13 presents live/dead staining images of L929 and HUVEC cells co-cultured with the extract solutions from each microparticle group for 1 and 3 days, all using a 1/16 concentration extract solution. The results demonstrate that most cells exhibited green fluorescence after co-culturing with the extract solutions, indicating good cell viability, morphology and uniform distribution. Red fluorescence representing dead cells was rarely observed, suggesting excellent biocompatibility and low cell toxicity within each group of particles.

**Figure 3.**
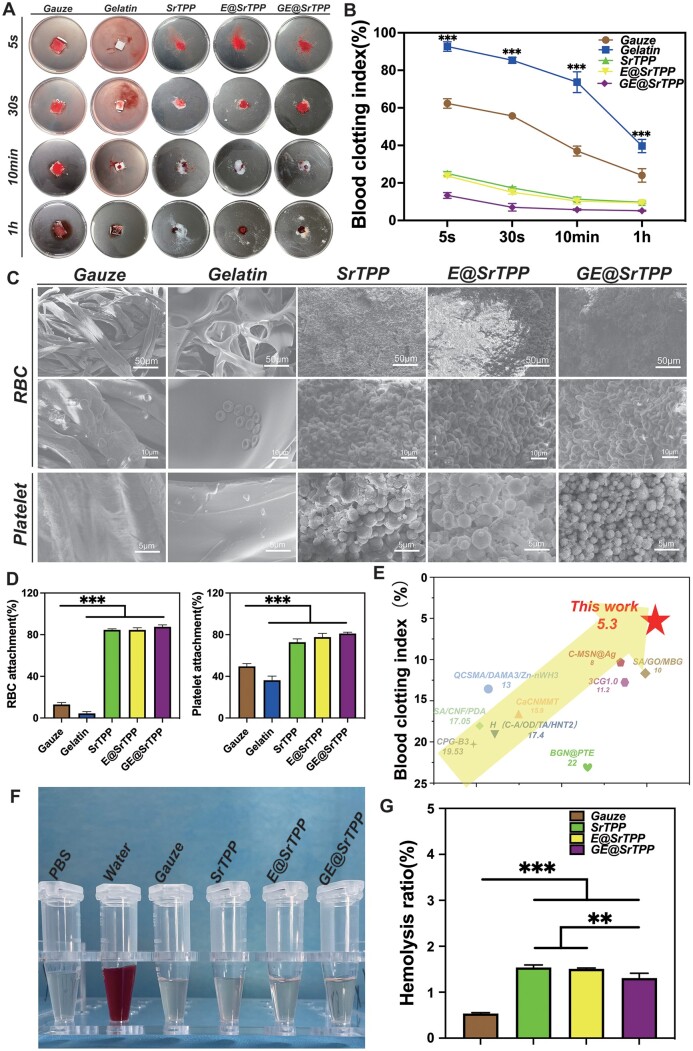
Hemostatic efficiency of GE@SrTPP. (**A**) Digital images from the BCI experiment; (**B**) BCI values at different time points; SEM images (**C**) and quantitative data (**D**) were obtained for each group of hemostatic materials to observe their induction of red blood cell and platelet aggregation. (**E**) The ashby diagram for the *in vitro* hemostatic efficiency of the previously reported hemostatic materials, compared to GE@SrTPP [18, 49–56]; digital images (**F**) and statistical data (**G**) of hemolysis experiments (*P < 0.05, **P < 0.01, ***P < 0.001).

To assess the hemostatic efficacy of each microparticle group in early-stage skin wound injuries, BCI experiments were conducted using commercialized gelatin hemostatic sponge and gauze as control materials. The absorption rate of hemoglobin at different time points (5 seconds, 30 seconds, 10 minutes, 1 hour) was compared to determine blood clotting efficiency. As depicted in Figure 3A, digital images from the BCI experiment reveal that the washing solution of gauze and gelatin sponge groups displayed deeper red coloration after contacting blood droplets for 30 seconds followed by washing with PBS; this indicates difficulty inducing RBCs aggregation and an abundance of free RBCs within these materials. In contrast, GE@SrTPP showed lighter or even transparent washing solution colors along with lower BCI values (5.4%), signifying superior hemostatic performance compared to other groups including medical gauze and commercialized gelatin sponge. As shown in Figure 3B, we conducted a quantitative comparison of the absorption rates of hemoglobin solutions (BCI values) at various time points (5 seconds, 30 seconds, 10 minutes, 60 minutes). The results indicated that the BCI value of GE@SrTPP remained relatively constant across each time point, providing further evidence for the remarkably rapid solidification capability of GE@SrTPP. Furthermore, the interaction between microparticles from each group, RBCs, and platelets can be observed through SEM images shown in Figure 3C. Only a small amount of blood cells adhered to gauze without forming aggregates while numerous RBCs and platelets were aggregated with the microparticles of each group, forming structures similar to blood clots. The SEM images also reveal that the platelets in the GE@SrTPP group were deformed and extended many pseudopodia, and secreted fibrinogen clusters, indicating that GE@SrTPP significantly induces platelet activation and fibrinogen production, which may be attributed to the Ga ions-induced production of thrombin and fibrinogen [27]. As shown in Figure 3D, a quantitative analysis of the adhesion of platelets and erythrocytes, SrTPP (72.8 ± 3.1%, 84.6 ± 1.0%), E@SrTPP (77.7 ± 3.6%, 84.6 ± 2.2%), GE@SrTPP(81.1 ± 1.3%, 87.6 ± 1.8%), also confirms that GE@SrTPP exhibits superior ability in aggregating blood cells compared to gauze and gelatin. The presence of dopamine in the microparticles, particularly its positive charge characteristic, facilitates ion adsorption and enhances RBC aggregation. In conclusion, our data confirms that GE@SrTPP achieves rapid hemostasis through the following process: firstly, it absorbs body fluid via mesoporous morphology and ion adsorption action, thereby accelerating RBC aggregation and adhesion to initiate the hemostasis process. Additionally, gallium ions activate platelets to trigger the coagulation cascade, promoting the formation of blood clots and establishing a physical barrier.

In summary, GE@SrTPP demonstrated both rapid and efficient hemostasis. Comparative analysis of the relevant BCI values and clotting times of GE@SrTPP with those of previously reported hemostatic materials was conducted. The Ashby diagram in Figure 3E illustrates the superiority of GE@SrTPP in achieving rapid and efficient hemostasis, establishing it as an outstanding hemostatic material compared to previously reported materials. Notably, the inclusion of Ga element significantly enhanced the hemostasis efficiency of SrTPP by promoting blood cell aggregation function and activating the coagulation process. Furthermore, blood compatibility assessment was performed on the particles. As depicted in Figure 3F and G, after co-culturing with blood for 1 hour, supernatants of SrTPP, E@SrTPP and GE@SrTPP remained clear and transparent similar to medical gauze. Hemolysis rate measurements at 540 nm revealed low rates for all groups: gauze (0.54 ± 0.02%), SrTPP (1.54 ± 0.06%), E@SrTPP (1.51 ± 0.02%) and GE@SrTPP (1.31 ± 0.10%). These results indicate that all groups exhibited minimal hemolysis rates well compliant with the international standard for hemostatic material [57]. The remarkable hemostatic effect of GE@SrTPP may be attributed to a synergistic combination of multiple factors. Firstly, the mesoporous structure of the material facilitates rapid water absorption for blood concentration, while SrTPP particles promote adhesion of RBCs and platelets, as well as blood cluts formation. Furthermore, with the modification of MPNs, Ga ions can further activate coagulation reactions and achieve robust hemostasis. Although Sr^2+^ have been reported to activate platelets and thrombin in initiating hemostatic reactions, they are unable to fully replace the role of calcium ions [58]. This study has demonstrated that SrTPP exhibits superior hemostatic efficiency compared to gauze and gelatin sponge, possibly due to its rapid water absorption properties and limited coagulation activation by Sr ions [58, 59]. Despite the relatively low content of Ga element in GE@SrTPP, it significantly enhances the microparticle’s hemostatic effect through its ability to activate intrinsic coagulation pathways.

### The antibacterial properties of GE@SrTPP

**Figure 4.**
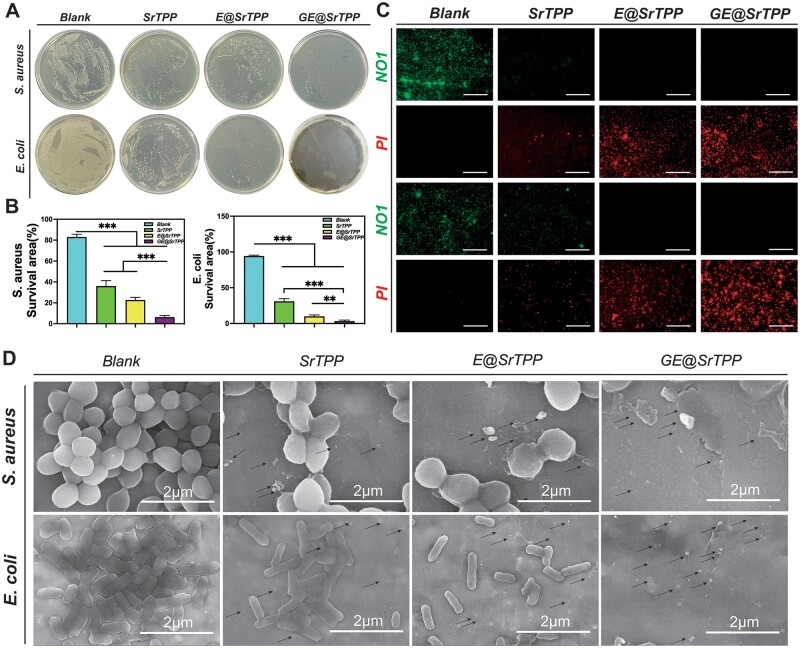
The antibacterial properties of GE@SrTPP. The antibacterial capacity of the microparticles against *Staphylococcus aureus* (*S. aureus*) and *Escherichia coli* (*E. coli*) was detected by counting the colony numbers on agar plates. The digital images (**A**), quantitative data (**B**) show the growth of bacteria on a plate after co-culturing with microparticles. (**C**) Fluorescence images of bacterial live/dead staining following co-culture with microparticles. (**D**) SEM images depicting the morphology of bacteria post co-culture with microparticles.

The antibacterial activity of the hemostatic powder can minimize the infection of harmful bacteria and improve wound healing; therefore, it is crucial to evaluate the *in vitro* antibacterial capacity of the microparticles. In this study, the antibacterial capacity of the microparticles against *S. aureus* and *E. coli* was detected by counting the colony numbers on agar plates. As shown in Figure 4A and B, SrTPP exhibits limited antibacterial capacity, with the remaining areas for *S. aureus* and *E. coli* being 36.0 ± 5.3% and 31.0 ± 3.6%, respectively. After being modified by EGCG, E@SrTPP significantly improved the antibacterial capacity, while after being modified by MPNs, GE@SrTPP exhibited the optimal antibacterial capacity, with the remaining areas for *S. aureus* and *E. coli* being only 6.3 ± 1.5% and 3.3 ± 1.5%, respectively. The bacterial live/dead staining photographs after co-culture with each group of particles also confirmed this, as shown in Figure 4C. There were a large number of red fluorescently marked dead bacteria in the GE@SrTPP group, which fully proved the antibacterial capacity of GE@SrTPP. The SEM images (Figure 4D) showed the morphology of the bacteria after co-culture, and the bacteria in the GE@SrTPP group showed a morphology of damaged bacterial membrane and shrinking bacteria. The antibacterial properties of GE@SrTPP have met expectations compared to previous materials (Supplementary Table S3). Ga ions significantly enhance the antibacterial effect of SrTPP, as it competes with iron ions in bacteria to bind to iron receptor proteins, causing disruption of bacterial iron metabolism and bacterial death [27].

### *In vivo* hemostasis performance of GE@SrTPP

**Figure 5.**
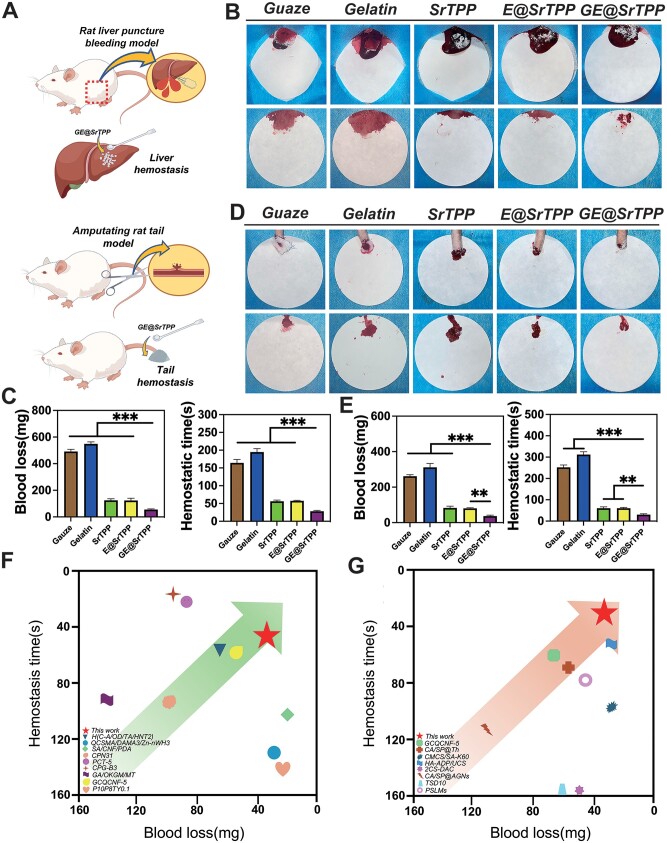
*In vivo* hemostasis performance of GE@SrTPP. (**A**) Schematic image of the modeling process for liver puncture and tail-amputation bleeding models. (**B**–**E**) Digital images and quantitative data on bleeding time and volume of microparticles applied to liver puncture models (**B** and **C**) and tail-amputation models (**D** and **E**) for hemostasis; the ashby diagrams for the hemostatic efficiency in rat liver puncture (**F**) and tail-amputation models (**G**) of the previously reported hemostatic materials, compared to GE@SrTPP [49, 50, 52, 53, 62–71, 72–75] (*P < 0.05, **P < 0.01, ***P < 0.001).

In order to evaluate the *in vivo* hemostasis ability of GE@SrTPP, a rat liver trauma and tail-amputation model was employed for validation (Figure 5A). As shown in Figure 5B and C, in the liver trauma model, the gauze and medical sponge groups showed the most bleeding volume and longer bleeding time (respectively, 491.1 ± 16.5 mg, 164.0 ± 9.8 seconds and 548.7 ± 15.3 mg, 194.7 ± 9.7 seconds). In addition, SrTPP, E@SrTPP particles showed certain hemostasis characteristics, while GE@SrTPP showed the least bleeding volume (54.9 ± 5.2 mg) and the shortest bleeding time (28.0 ± 2.6 seconds). As shown in Figure 5D and E, in the tail-amputation model, compared with other groups, GE@SrTPP group also showed the least bleeding volume (36.8 ± 5.6 mg) and the shortest bleeding time (30.0 ± 4.63 seconds). This result is consistent with the previous data [32], SrTPP can absorb plasma quickly in the initial bleeding and induce RBCs and platelet aggregation to form a blood clot to seal the wound. However, the physical barrier formed by SrTPP cannot quickly suppress bleeding, resulting in a small amount of blood flowing out. After modification by MPNs, the hemostasis ability of SrTPP was significantly enhanced. In particular, after modification by MPNs, GE@SrTPP showed the best hemostasis efficiency, indicating that EGCG, Ga and SrTPP formed a synergistic effect by aggregating RBCs and platelets on the wound surface to quickly seal the wound and further activate platelets to prevent internal tissue from bleeding further [32,60,61]. According to the Ashby diagram shown in Figure 5F and G, compared with previously reported materials (Supplementary Table S4), GE@SrTPP exhibits superior hemostatic ability, with lower bleeding volume and shorter bleeding time in rat liver trauma and tail-amputation models than other hemostatic materials. Therefore, in terms of hemostatic powder alone, GE@SrTPP has good potential for clinical emergency hemostasis treatment.

### Anti-inflammatory, antioxidant and angiogenic capabilities of GE@SrTPP

**Figure 6.**
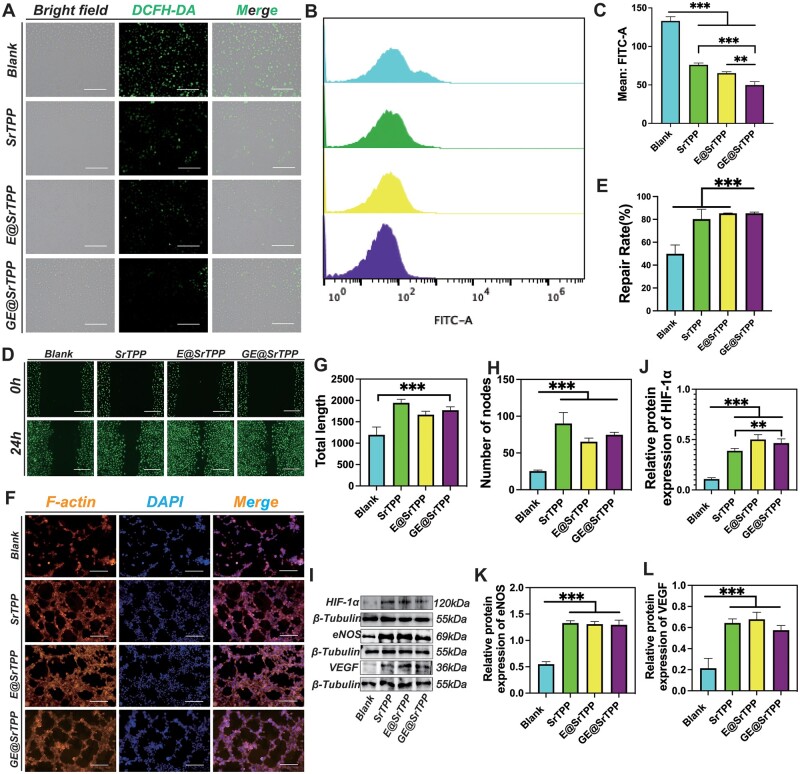
Antioxidant, and angiogenic capabilities of GE@SrTPP. (**A**–**C**) The capability of GE@SrTPP for alleviate oxidative stress using the ROS fluorescent probe DCFH-DA. Experimental data including (**A**) fluorescence images of ROS in each cell group labeled with DCFH-DA, (**B**) flow cytometry analysis and (**C**) quantitative data; (**D** and **E**) fluorescence images and quantitative data of scratch experiments based on HUVECs; (**F**–**H**) experimental data on HUVECs tube formation in MatriGel, including (**F**) fluorescence images, (**G**) quantification of total tube length, and (**H**) quantitative analysis of nodes. (**I**–**L**) Western blot analysis of the effect of microparticles on the expression levels of angiogenesis-related proteins in HUVECs, including (**I**) western blot bands, and (**J**–**L**) quantitative data of related proteins (*P < 0.05, **P < 0.01, ***P < 0.001) (bar = 200 μm).

Bleeding and infection are early events in skin injury, followed by the inflammatory and proliferative phases. During the inflammatory phase of wound healing, excessive ROS lead to oxidative stress in the wound, and uncontrolled inflammatory responses hinder the normal fibroblast cells from participating in the wound-healing process [76, 77]. Subsequently, we assessed the ability of GE@SrTPP to alleviate oxidative stress using the classical ROS fluorescent probe DCFH-DA. As depicted in Figure 6A, the GE@SrTPP group exhibited the lowest level of green fluorescence (ROS) after hydrogen peroxide stimulation, which was further confirmed by flow cytometry data (Figure 6B and C). The results showed that GE@SrTPP significantly reduced ROS levels in cells. Similarly, SrTPP and E@SrTPP groups also demonstrated a significant reduction in ROS levels due to their rich catechol groups that can be oxidized by ROS into quinones for effective elimination [78].

To evaluate the regulatory role of microparticles in wound healing during the proliferation stage, this study assessed the effects of each group of microparticles on endothelial cell migration using an *in vitro* cell migration assay. As shown in Figure 6D and E, SrTPP, E@SrTPP and GE@SrTPP groups all showed a promoting effect on cell migration, with migration rates of 80.2 ± 8.6%, 85.3 ± 0.2% and 85.3 ± 1.1% (Figure 6E), respectively, within 24 hours. Furthermore, as illustrated in Supplementary Figure S14, these particles enhance the migration of fibroblasts, a process that is essential for expediting wound healing. The groups of strontium-containing microparticles all demonstrated a preferential enhancement of L929 cell migration, aligning with prior research indicating that strontium ions promote fibroblast migration to facilitate wound healing [79]. There was no significant difference among these three groups. Furthermore, the study evaluated the angiogenic effects of microparticles on HUVECs using an *in vitro* tube formation assay. As shown in Figure 6F, SrTPP, E@SrTPP and GE@SrTPP groups all showed more tubular network formation. Quantitative data also confirmed this, as the capillary lengths (Figure 6G) (SrTPP, E@SrTPP and GE@SrTPP groups were 1942.0 ± 84.1 μm, 1666.0 ± 80.9 μm and 1770.3 ± 81.6 μm, respectively) and nodes (Figure 6H) (SrTPP, E@SrTPP and GE@SrTPP groups were 90.0 ± 15.0, 65.3 ± 4.9, 74.7 ± 3.5, respectively) of these three groups were significantly better than the blank group. Additionally, Figure 6I shows the expression levels of angiogenesis-related (eNOS, HIF-1a, VEGF) proteins in HUVECs co-cultured with each group of microparticles extract solution (dilution ratio: 1/16) for 3 days. The results show that the expression levels of eNOS, HIF-1a and VEGF proteins were significantly increased in all groups of microparticles (Figure 6J–L), indicating that GE@SrTPP can boost VEGF expression and angiogenesis by activating the HIF-1α/VEGF signaling pathway in HUVECs [80–82]. These data mean that microparticles are expected to play a role in promoting cell migration and angiogenesis during wound healing in the proliferative stage, which is crucial for accelerating wound healing [83]. Based on the data analysis, there was no statistically significant difference in the angiogenic effects of SrTPP, E@SrTPP and GE@SrTPP, suggesting that the release of strontium ions from the microparticles played a predominant role. Previous reports have indicated that Sr^2+^ primarily exerts its skin regeneration effects through two pathways. Firstly, as an effective angiogenic bioactive metal ion, Sr ions can stimulate angiogenesis of endothelial cell, thereby reconstructing damaged blood circulation at the wound site with newly formed blood vessels [26, 84, 85]. Secondly, Sr ions can facilitate cell migration and proliferation to expedite wound healing [86, 87]. This suggests that following hemostasis and anti-inflammation stages, GE@SrTPP can continue to exert its influence during the wound-healing stage by releasing Sr ions to accelerate full-thickness skin injury recovery.

**Figure 7.**
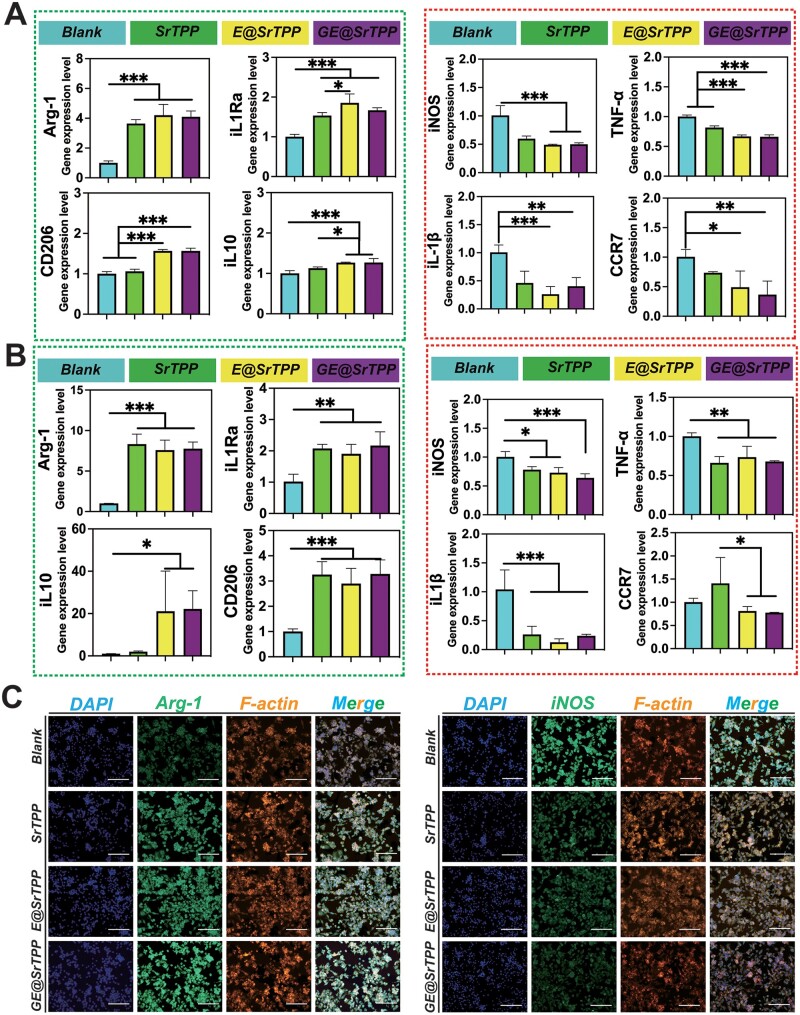
The effect of GE@SrTPP on the M1/M2 polarization of macrophage *in vitro*. (**A**) Q-PCR analysis was proposed to detect the expression of M1/M2 polarization-related genes in Raw264.7 after 3 days of culture. (**B**) Q-PCR analysis was proposed to detect the expression of M1/M2 polarization-related genes in Raw264.7 after 7 days of culture. (**C**) Microscopic fluorescence images showed the iNOS levels and arg-1 levels in Raw264.7 of each group (bar = 200 μm) (*P < 0.05, **P < 0.01, ***P < 0.001).

In addition, to assess the regulatory effect of GE@SrTPP on macrophage polarization, RAW264.7 cells were co-cultured with extracts of each microparticle for 3 and 7 days under LPS stimulation. Q-PCR results revealed the expression levels of pro-inflammatory and anti-inflammatory genes in Raw264.7 cells for each microset at different time points (Figure 7A and B). The findings indicated that the SrTPP group exhibited a certain degree of anti-inflammatory regulatory effect compared to the blank group at 3 days. Conversely, E@SrTPP and GE@SrTPP demonstrated significantly superior anti-inflammatory regulatory effects than SrTPP. After 7 days, the SrTPP, E@SrTPP and GE@SrTPP group still displayed a certain level of anti-inflammatory regulatory ability compared to the blank group. This suggests that dandelion-like particles possess potent inflammatory regulatory effects. As shown in Supplementary Figure S15, EGCG is predominantly released in the early stages of inflammation, whereas dopamine exhibits minimal early release but maintains a stable long-term release profile. This behavior can be attributed to strong electrostatic interactions between positively charged dopamine and strontium ions along with triphosphate ions. Notably, there are distinct differences in how EGCG and dopamine contribute to various processes involved in inflammation regulation. Immunofluorescence staining images further confirmed this observation as Raw264.7 cells in SrTPP, E@SrTPP and GE@SrTPP groups displayed significantly lower iNOS protein levels and higher Arg-1 protein levels compared to the control group (Figure 7C). Given that iNOS and Arg-1 are hallmark proteins for Ml polarization and M2 polarization of macrophages respectively [88, 89], the aforementioned data validate that GE@SrTPP can drive macrophage polarization toward M2 phenotype while inhibiting pro-inflammatory phenotypes during skin wound inflammation phase, and also contribute to ROS scavenge and oxidative stress reduction. ROS can exacerbate severe inflammation by rapidly activating signal transduction pathways that lead to the production of inflammatory cytokines [90]. Previous results have demonstrated that GE@SrTPP particles possess a remarkable capacity for intracellular ROS clearance, which is essential for modulating the inflammatory microenvironment and facilitating M2 polarization of macrophages.

### *In vivo* antimicrobial properties of GE@SrTPP

We further assessed the antimicrobial activity of GE@SrTPP *in vivo* using an infected full-layer skin wound model. On the third day, visible yellow pus was observed around the wound in the control group, as well as some suppuration in SrTPP, E@SrTPP and GE@SrTPP (Supplementary Figure S16A). The number of bacterial colonies provided strong evidence for this observation. By the seventh day, bacterial colonies were significantly reduced in SrTPP and E@SrTPP groups, while almost no bacteria were present in the GE@SrTPP group (Supplementary Figure S16D and E). As depicted in Supplementary Figure S16B and C, GE@SrTPP demonstrated superior wound healing compared to other groups. These findings confirm that the application of GE@SrTPP can greatly enhance the healing of infected wounds. Furthermore, the wound-healing effect of GE@SrTPP was evaluated using H&E staining, Masson’s trichrome staining, TNF-α and IL-1β analysis (Supplementary Figure S16F–H). On day 3, inflammation was evident across all groups; however, it was relatively mild in E@SrTPP and GE@SrTPP groups. By day 7, inflammation remained significant in the control group but showed a notable decrease in both E@SrTPP and GE@SrTPP groups. In addition, HE staining of organs in each group of models (Supplementary Figure S17) revealed that after 14 days of treatment with microparticles, no lesions or injuries were observed in the heart, liver, spleen, lungs or kidneys of the rat models. These findings indicate the favorable biological safety profile of this dandelion-like microparticle.

### *In vivo* wound-healing effect

**Figure 8.**
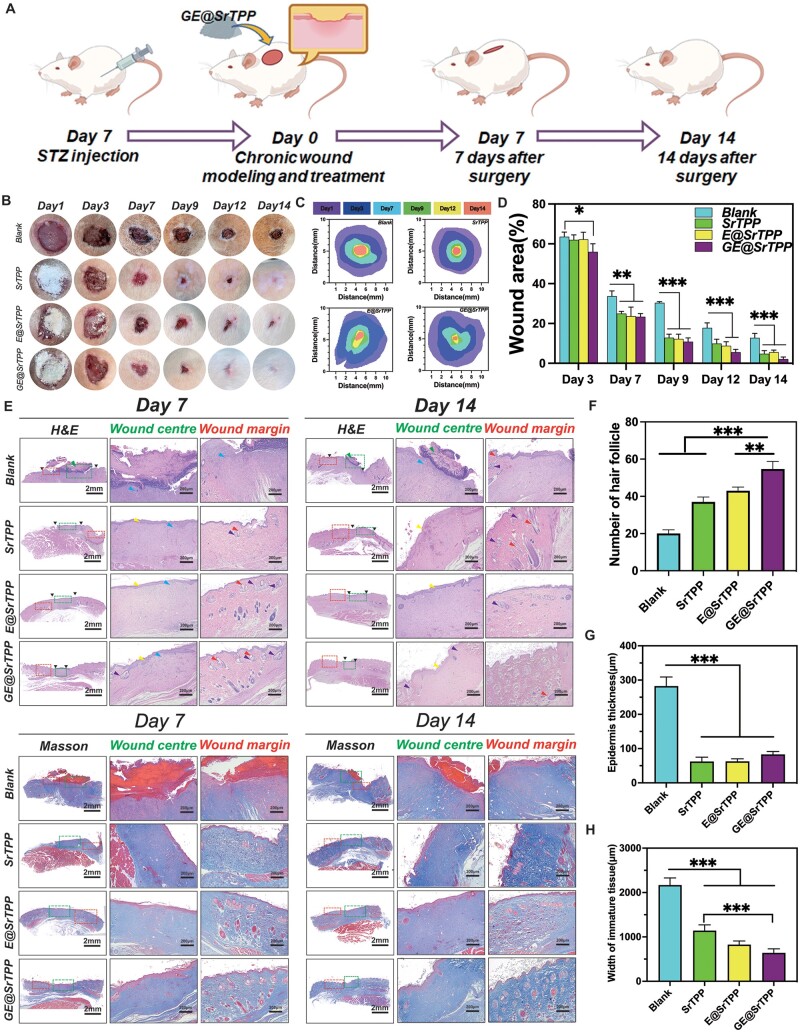
GE@SrTPP Promoted chronic full-thickness wound repair and regeneration *in vivo*. (**A**) Schematic image of modeling procedure for rat chronic full-thickness wounds. (**B** and **C**) Representative images (**B**) and schematic images of wound area (**C**) of the wounds in response to microparticles on days 1, 3, 7, 9, 12 and 14. (**D**) Quantitative analysis of wound-healing rate for each group. (**E**) H&E and Masson’s staining of wound tissues obtained from various groups on days 7 and 14 (*n* = 3, *P < 0.05, **P < 0.01, the margin and center of the wound were marked with red and blue boxes, respectively). Yellow, green, blue, red and purple arrows represented epithelial layer, scabs, inflammatory infiltration, hair follicles and adjacent sebaceous glands, respectively. Quantitative statistics of the number of hair follicle (**F**), epidermal thickness (**G**) and width of immature tissue (**H**) (*n* = 3, *P < 0.05; **P < 0.01; ***P < 0.001).

**Figure 9.**
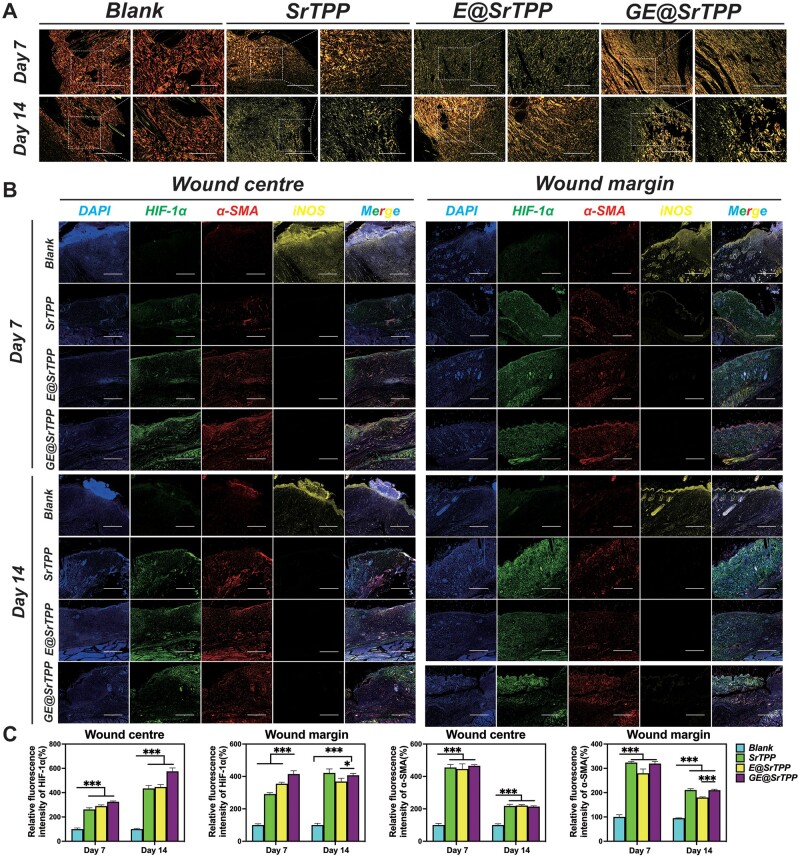
(**A**) Sirius red staining indicated the collagen arrangement in all groups; (**B**) immunofluorescence staining images and their quantitative data for the protein levels related to angiogenesis (HIF-1α and α-SMA) and inflammation (iNOS) in the regenerative skin tissues of each group (bar = 200 μm, *n* = 3, *P < 0.05; **P < 0.01; ***P < 0.001).

In order to verify the *in vivo* wound-healing effect, this study assessed the therapeutic effects of each group of microparticles using a full-thickness skin chronic defect model. The entire animal experiment procedure is presented in Figure 8A. Firstly, diabetic rats (type I) were induced by injection of Streptozotocin (STZ), followed by creation of an 8-mm full-thickness skin wound on the back of the rats. GE@SrTPP was then utilized as a wound dressing and evenly applied to the wound. Representative pictures and schematic diagrams of the wound area at different time points after treatment with each group of particles are shown in Figure 8B and C. On the third day, pus scabs formed on the wound bed of the blank group. No pus scabs were observed on the wound bed of E@SrTPP and GE@SrTPP groups. Uncontrolled wound infection can seriously impede the healing process because few physical barriers are formed. GE@SrTPP can effectively create a physical barrier on the defective part of the wound, due to the release of Ga^3+^. The wound-healing rate of the control group (66.3%) was significantly lower than that of the SrTPP (75.0%), E@SrTPP (76.4%) and GE@SrTPP (76.4%) groups as depicted in Figure 8D after 7 days of treatment. Compared to other groups, the GE@SrTPP group exhibited superior efficacy in promoting wound healing after 14 days of treatment (97.9%), with almost complete disappearance of the wound. Previous data have confirmed that GE@SrTPP plays a role in accelerating wound healing by modulating inflammation, scavenging ROS, promoting cell proliferation and angiogenesis during the inflammatory and proliferation stages. Compared to the blank group, there was a lower and more irregular number of new capillaries, while SrTPP, E@SrTPP and GE@SrTPP showed a significantly higher and more uniform number of new capillaries (Supplementary Figure S18). To further investigate the effects of GE@SrTPP on tissue remodeling during wound healing, histological staining was performed on tissue surrounding the wound site (Figure 8E). On day 7 post-treatment, histological analysis using H&E staining and Masson’s staining revealed that the wound center in the GE@SrTPP-treated group had undergone re-epithelialization. In contrast, the other groups exhibited less newly formed tissue and looser connective tissue. Notably, the blank group still presented visible scabs on the wound surface with higher levels of inflammatory infiltration. Over time, most wounds achieved complete epithelial coverage, particularly in the GE@SrTPP-treated group, where a greater abundance of regenerated hair follicles and adjacent sebaceous glands were observed. Quantitative analysis demonstrated that the GE@SrTPP group exhibited a higher number of regenerated hair follicles compared to other groups (Figure 8F). The epidermal thickness of GE@SrTPP is 83.0 ± 8.5 μm, which is significantly thicker than that of SrTPP (62.0 ± 12.5 μm) and E@SrTPP (62.0 ± 7.5 μm) (Figure 8G). We have compared the effect of materials on wound closure and remodeling with those previously reported, and the results were also in line with expectations (Supplementary Table S5). At the same time, the width of the immature tissue in GE@SrTPP group was significantly narrower than that in the Blank group (Figure 8H). At the early stage (day 3), the expression of CD86, representing M1 phenotype macrophages in the E@SrTPP and GE@SrTPP groups, was significantly lower than that in the blank group. Conversely, the expression of CD206, representing M2 phenotype macrophages, was much higher in the E@SrTPP and GE@SrTPP groups compared to the blank group. Throughout the entire healing process, there was a gradual decrease in the expressions of TNF-α, IL-1β and CD86. The expressions of these markers were consistently lower in both E@SrTPP and GE@SrTPP groups compared to those in the blank group. However, there was a gradual increase in CD86 expression over time. These findings suggest that GE@SrTPP plays a role in reducing inflammation throughout the healing process (Supplementary Figure S19). Furthermore, polarized microscopy images of histological tissues stained with Sirius red indicated a more disordered collagen arrangement in the blank group as opposed to an oriented collagen fiber structure in the GE@SrTPP group (Figure 9A). These data suggest that GE@SrTPP significantly promotes chronic skin wound regeneration, resulting in a satisfactory recovery. In addition, HE staining of organs in each group of models (Supplementary Figure S20) revealed that after 14 days of treatment with microparticles, no lesions or injuries were observed in the heart, liver, spleen, lungs or kidneys of the rat models. These findings indicate the favorable biological safety profile of this dandelion-like microparticle.

In this study, immunofluorescence staining was conducted on skin tissue samples collected at postoperative days 7 and 14 to assess the *in vivo* angiogenesis and inflammation regulatory effects of GE@SrTPP (Figure 9B). The results revealed that the blank group exhibited strong iNOS signal positivity, indicating uncontrolled severe inflammation. Lower levels of iNOS protein were observed in the SrTPP, E@SrTPP and GE@SrTPP groups. New vessel formation serves as a reliable indicator of wound-healing efficacy. Fluorescence staining and corresponding quantitative analysis demonstrated that the GE@SrTPP group displayed the highest levels of HIF-1α at both the wound margin and center on day 7 and day 14 after treatment. Compared to the blank group, SrTPP, E@SrTPP and GE@SrTPP groups all showed high levels of α-SMA protein, and there was no significant difference between the three groups, whether in the center or edge of the wound, indicating that Sr^2+^ play a dominant role in wound angiogenesis. This is consistent with the above results, Sr^2+^ released by GE@SrTPP activated HIF-1α/VEGF signaling cascade to enhance wound angiogenesis. CD31 immunohistochemical staining further confirmed that GE@SrTPP could enhance angiogenesis levels to promote skin wound healing (Supplementary Figure S21). In conclusion, these findings indicate excellent performance of GE@SrTPP in promoting full-thickness skin wound healing.

Considering the sequential and overlapping stages of skin tissue response to injury, we designed a dandelion-shaped Sr–Ga particle GE@SrTPP to comprehensively regulate wound healing at different stages. In the early stage of skin injury, the mesoporous structure of GE@SrTPP facilitates rapid absorption of bodily fluids and enhances hematocrit level to promote blood cell aggregation. Ga^3+^ released by GE@SrTPP further enhances its function by activating platelets and increasing fibrinogen production to synergistically promote blood coagulation. Previous studies have demonstrated that platelet-induced stress during blood clot maturation drives clot contraction, which is believed to facilitate wound closure [91]. More significantly, released Ga^3+^ outcompete iron ions in bacterial iron receptor proteins, leading to disruption of bacterial iron metabolism and subsequent bacterial death. Our data also confirm that Ga-MPNs enhance the antibacterial activity of SrTPP, allowing GE@SrTPP to achieve rapid hemostasis for wound sealing and prevention of bacterial infection from the external environment during the early-stage of wound healing. Based on a rat liver puncture and tail-amputation bleeding model, we further confirm that compared to most currently reported hemostatic materials, GE@SrTPP achieves high hemostasis speed and efficiency. In the subsequent inflammatory stage, GE@SrTPP significantly downregulates expression levels of pro-inflammatory factors in macrophages, including TNF-α and IL-1β, while promoting polarization of macrophages toward an anti-inflammatory M2 phenotype with significant upregulation of anti-inflammatory gene expression. Furthermore, GE@SrTPP has the potential to ameliorate oxidative stress in the wound area by scavenging ROS and eliminating detrimental factors for wound healing. During the proliferation stage of wound healing, both SrTPP and E@SrTPP, as well as GE@SrTPP, can enhance HUVECs migration and angiogenesis *in vitro* while upregulating the expression levels of angiogenesis-related proteins such as VEGF, eNOS and Hif-1α. This suggests that GE@SrTPP can boost VEGF expression and angiogenesis by activating the HIF-1α/VEGF signaling pathway in HUVECs. *In vivo* experiments also validated this finding, with all microparticles significantly increasing the expression of angiogenesis markers and promoting blood vessel regeneration while exhibiting high levels of HIF-1α-positive signals. This further indicates that Sr^2+^ played a pivotal role in this process by activating the HIF-1α/VEGF signaling cascade to enhance wound angiogenesis, akin to dandelion seeds’ effect. Sr^2+^ act as ‘seeds’ to stimulate blood vessel growth similar to root systems providing ‘nutrients’ for wound healing. Lastly, during the tissue remodeling stage of wound healing in a rat chronic full-thickness skin defect model, GE@SrTPP notably expedited functional skin tissue regeneration evidenced by increased hair follicles and more mature aligned collagen fibers in regenerated skin. Previous studies have also confirmed the role of strontium ions in promoting hair follicle regeneration and collagen reshaping. Therefore, the dandelion-shaped Sr–Ga microparticle GE@SrTPP achieves hierarchically stimulation and comprehensive regulation of the wound-healing process. Most existing reports on wound dressings, such as silicate particles, CeO_2_ nanocatalyst composite materials, and natural polymer hydrogels, focus on enhancing specific aspects of wound-healing improvement, such as improving angiogenesis, reducing oxidative stress or reducing inflammation [50, 92, 93]. The advantage of GE@SrTPP lies in its multifunctional properties as a dressing with hierarchically stimulation function that integrates rapid hemostasis, antibacterial effects, inflammation regulation and skin tissue reconstruction into a single system to ultimately achieve rapid functional skin regeneration. In particular, the synthesis method of GE@SrTPP is based on the self-assembly of strontium ions and tris(phosphate) salts induced by positively charged dopamine hydrochloride. This environmentally friendly material synthesis strategy enables mass production of GE@SrTPP under mild conditions, which is advantageous as inorganic materials often require high-temperature sintering. In summary, GE@SrTPP exhibits potential for meeting various requirements in different stages of wound healing, rendering it a promising candidate for applications in wound healing.

## Conclusion

In conclusion, we have successfully developed a dandelion-biomimetic strontium-gallium-based GE@SrTPP dressing that integrates the regulatory functions necessary for the biological processes of skin tissue from injury to healing. This dressing not only achieves rapid hemostasis but also rebuilds the wound microenvironment by addressing inflammation, infection, and blood circulation, thereby accelerating the healing of full-thickness skin wounds. SrTPP is a new environmentally friendly and biocompatible wound dressing synthesized through biomineralization and self-assembly under mild conditions, making it conducive to large-scale and uniform preparation for practical clinical applications. The distinctive mesoporous architecture and ion adsorption properties of the material promote the rapid absorption of bodily fluids, resulting in blood cell aggregation, adhesion, and the initiation of coagulation reactions. The MPNs constructed using Ga^3+^ and EGCG enhance SrTPP’s hemostatic efficiency and ROS scavenging function while providing excellent anti-infection ability. More importantly, GE@SrTPP exhibits a strong synergistic effect between gallium ions and strontium ions. Gallium ions enhance hemostasis efficiency and antibacterial properties in the early stage of wound healing, while strontium ions contribute to subsequent wound healing. The release of strontium ions from GE@SrTPP promotes angiogenesis during the proliferation and remodeling stages by increasing levels of HIF-1α and VEGF, resembling the dispersal and rooting process of dandelion seeds. The newly formed blood vessels act as a root system for nutrient transportation, accelerating the healing of full-thickness wounds. Additionally, GE@SrTPP powder can effectively cover wound defects of various shapes to meet complex clinical needs. It is noteworthy that the combination of strontium ions and catechol groups in GE@SrTPP leads to a significant reduction in inflammation and oxidative stress at the wound site, thereby eliminating adverse factors for wound healing. This novel dandelion-biomimetic dressing, GE@SrTPP, holds promise as a candidate for repairing multi-stage wounds. The scheme involving cationic-induced strontium biomineralization, self-assembly+MPNs for biomaterials preparation may prove to be an effective strategy for enhancing tissue regeneration.

## Supplementary Material

rbae121_Supplementary_Data

## Data Availability

Data will be made available on request.
