# Supplementary material for: Dandelion-shaped strontium-gallium microparticles for the hierarchical stimulation and comprehensive regulation of wound healing
Source: Regen Biomater. 2024 Oct 18;11:rbae121. doi: 10.1093/rb/rbae121 (PMC11561401; doi:10.1093/rb/rbae121)
Supplement: rbae121_Supplementary_Data [file rbae121_supplementary_data.docx]

**Supporting Information**

Dandelion-shaped strontium-gallium microparticles for the hierarchical stimulation and comprehensive regulation of wound healing

Minrui Ji ^a,b,1^, Zaixin Yuan ^c,1^, Hongdong Ma ^b,1^, Xian Feng ^b^, Cong Ye ^b^, Lei Shi ^b^, Xiaodong Chen ^a,^*, Fei Han ^b,^*, Caichou Zhao ^a,^*

^a^ *Department of Dermatology, Affiliated Hospital of Nantong University, Medical School of Nantong University, Nantong 226001, China.*

^b^ *Department of Orthopaedics, Affiliated Hospital of Nantong University, Medical School of Nantong University, Nantong 226001, China.*

^c^ *Department of Respiratory and Critical Care Medicine, Affiliated Hospital of Nantong University, Medical School of Nantong University, Nantong 226001, China.*

^1^ *These authors contributed equally to this work.*

*Corresponding author.

Email: cczhao@cmu.edu.cn (C.Z.); feyhan50089@outlook.com (F.H.); dermatochen@ntu.edu.cn (X. C.)

**Figures:**


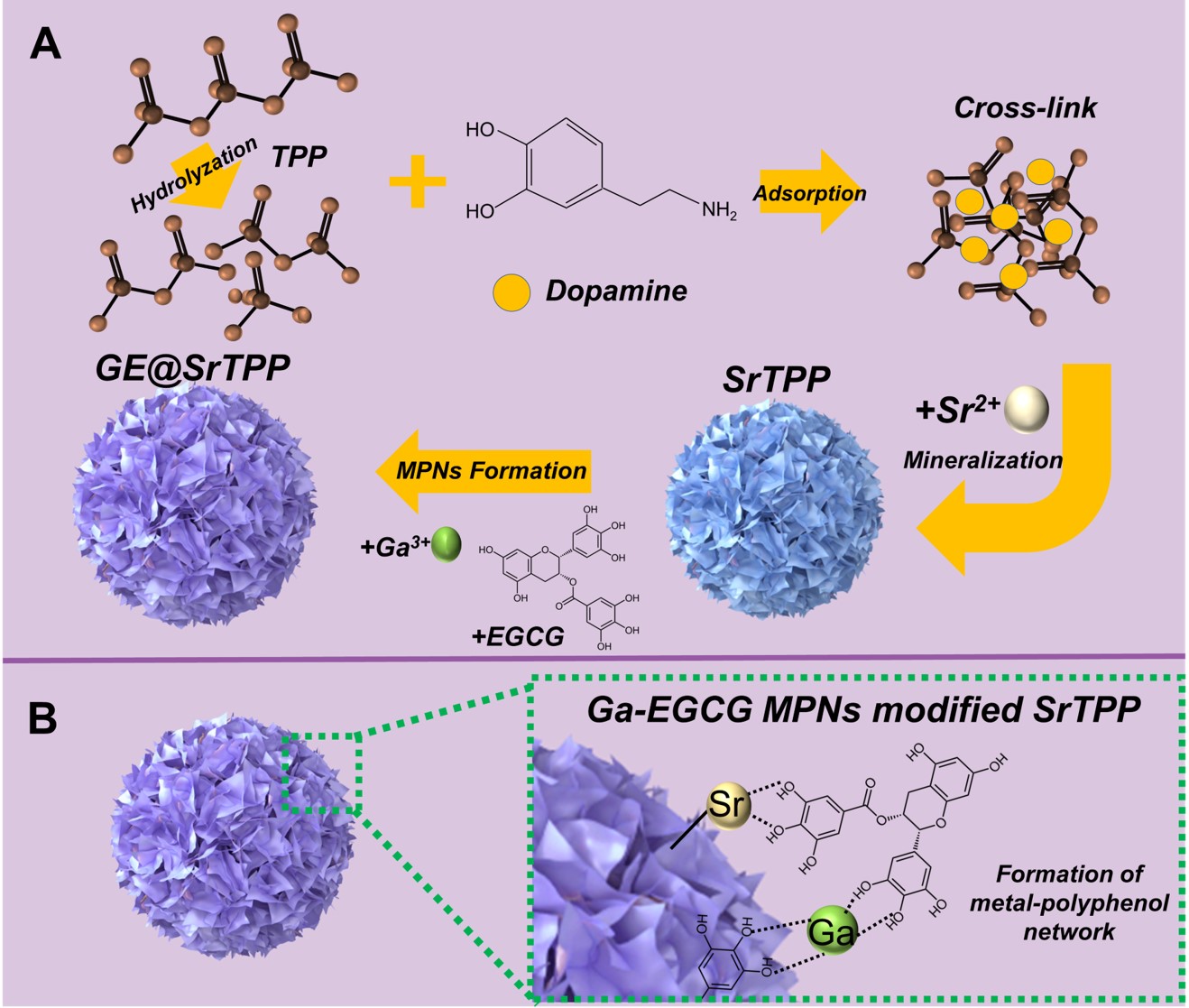


**Figure S1.** Schematic illustration of the principle of dandelion-shaped microparticles formation (A) and the following Ga-EGCG MPNs modification (B). The SrTPP was synthesized through the induction of dopamine, based on Sr ions and trisphosphate self-assembly. Furthermore, through the coordination of metal ions (Ga and Sr ions) with polyphenols, MPNs were incorporated with SrTPP.


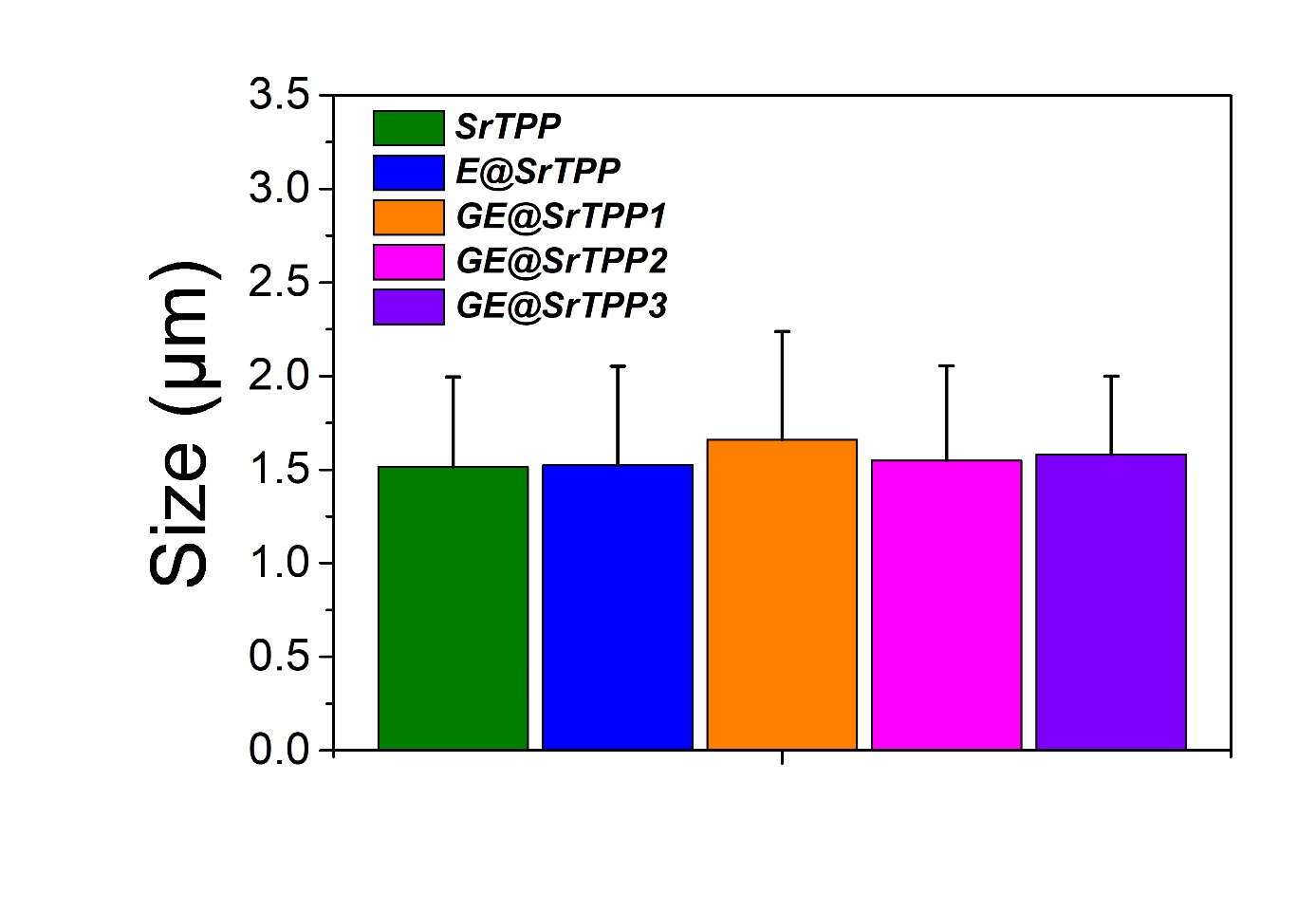


**Figure S2.** Quantitative data on the particle size of each group of microparticles.


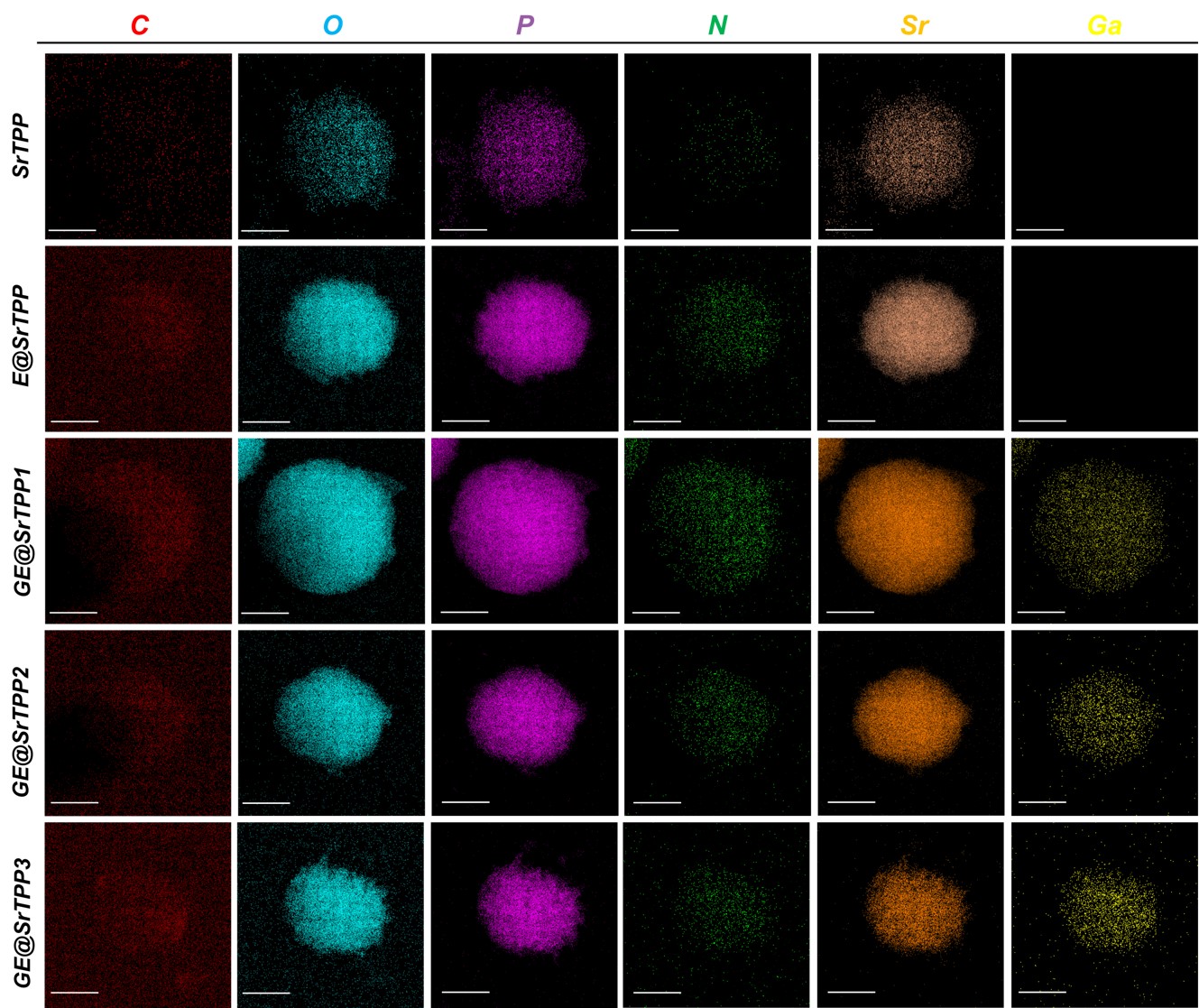


**Figure S3.** Mapping analysis of each group of microparticle.


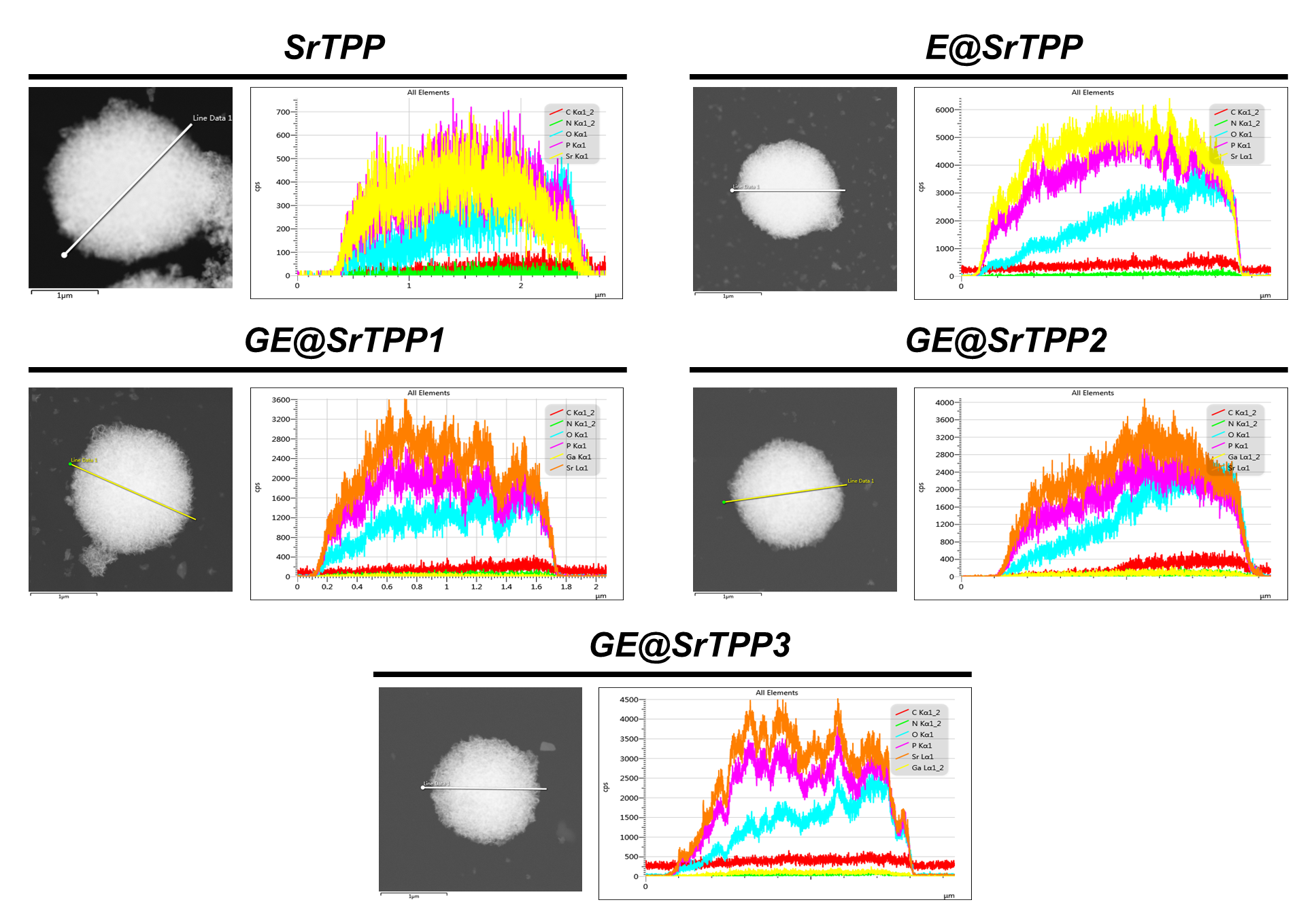


**Figure S4.** Line scan energy spectrum (EDS) analysis of each group of microparticle.


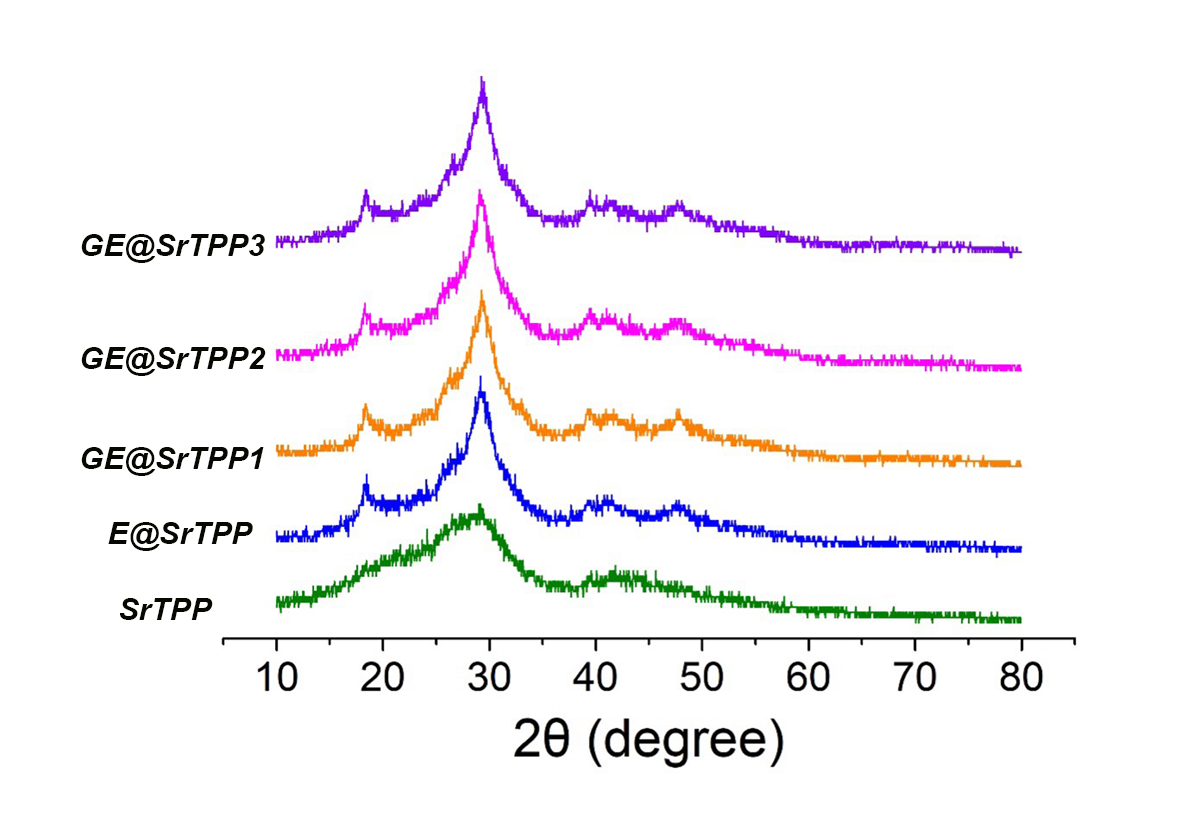


**Figure S5.** XRD analysis of each group of microparticles.


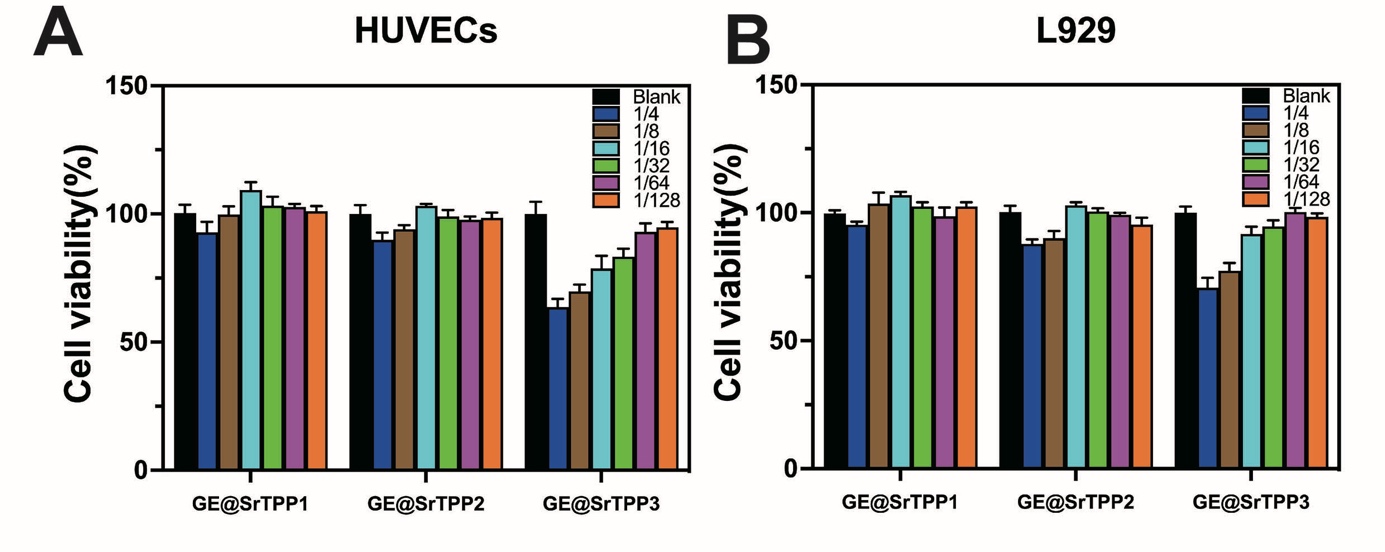


**Figure S6.** The biocompatibility of various GE@SrTPP particles. (A) CCK-8 analysis of the effect of extracts (different dilution ratios) of the GE@SrTPP particles on cell viability of human umbilical vein endothelial cells (HUVECs); (B) CCK-8 was ultilized to analyze the effects of the extracts (different dilution ratios) of the GE@SrTPP particles on the cell viability of the fibroblast cell line (L929).


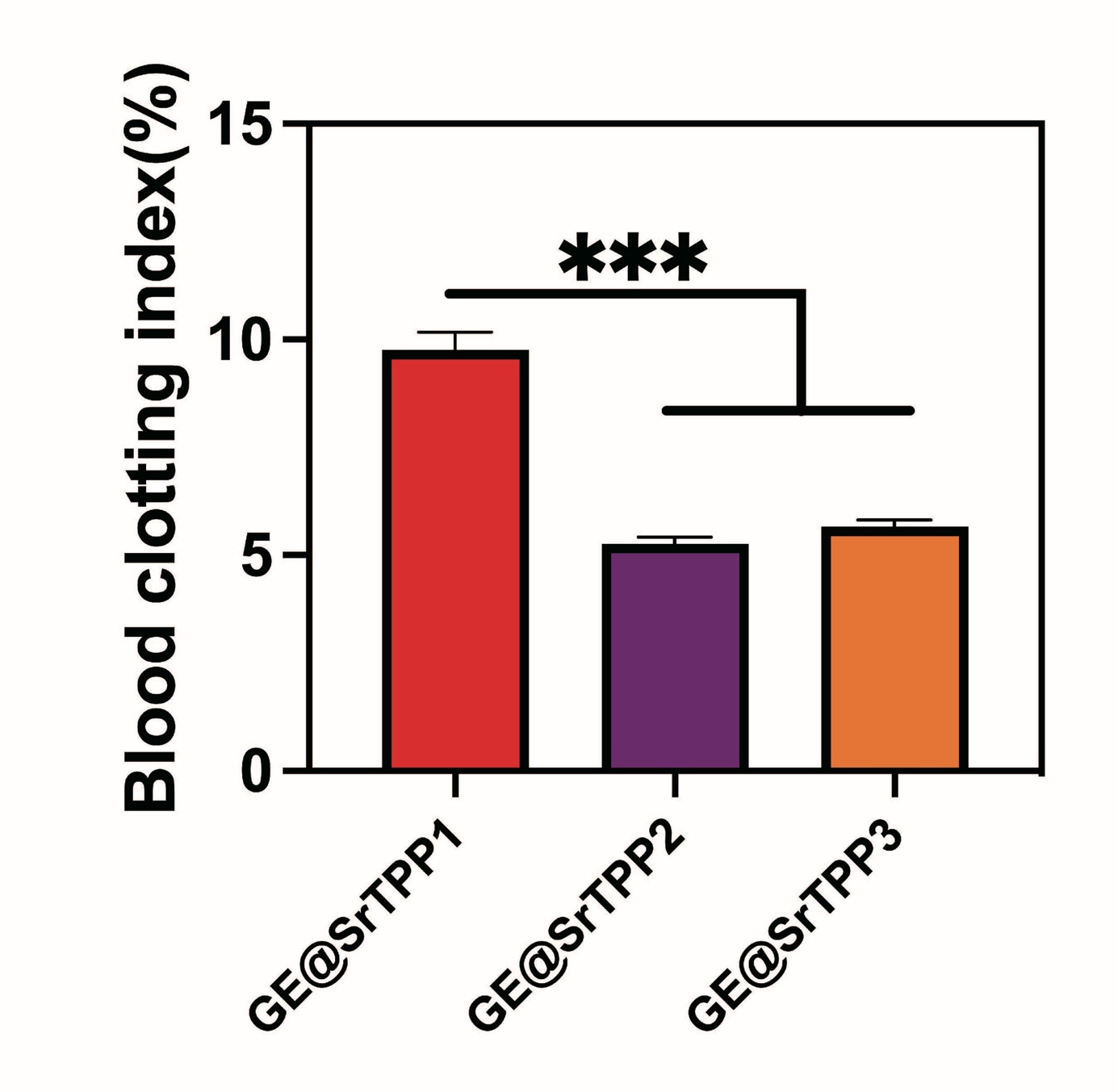


**Figure S7.** Preliminary exploration of the hemostasis efficiency of various GE@SrTPP microparticles based on blood clotting index (BCI) analysis. (* P < 0.05; ** P < 0.01; *** P < 0.001).


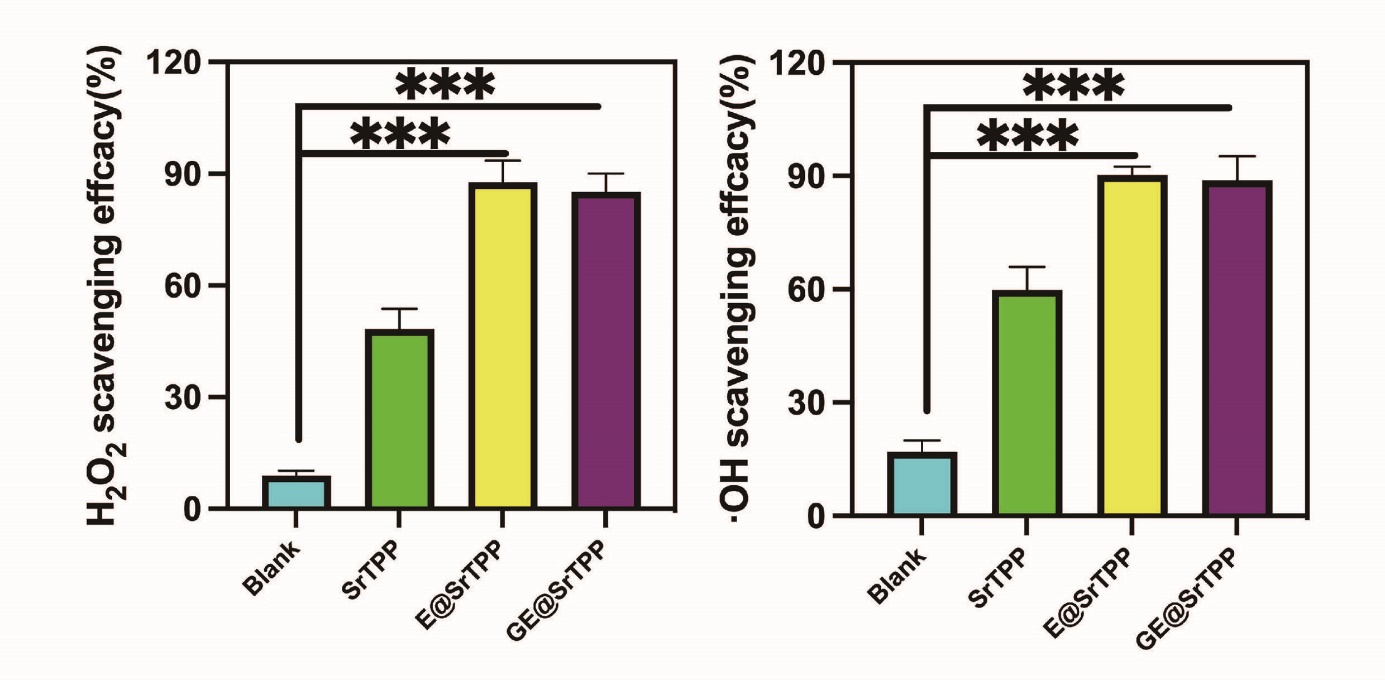


**Figure S8.** H_2_O_2_ and •OH scavenging efficiency of microparticles.


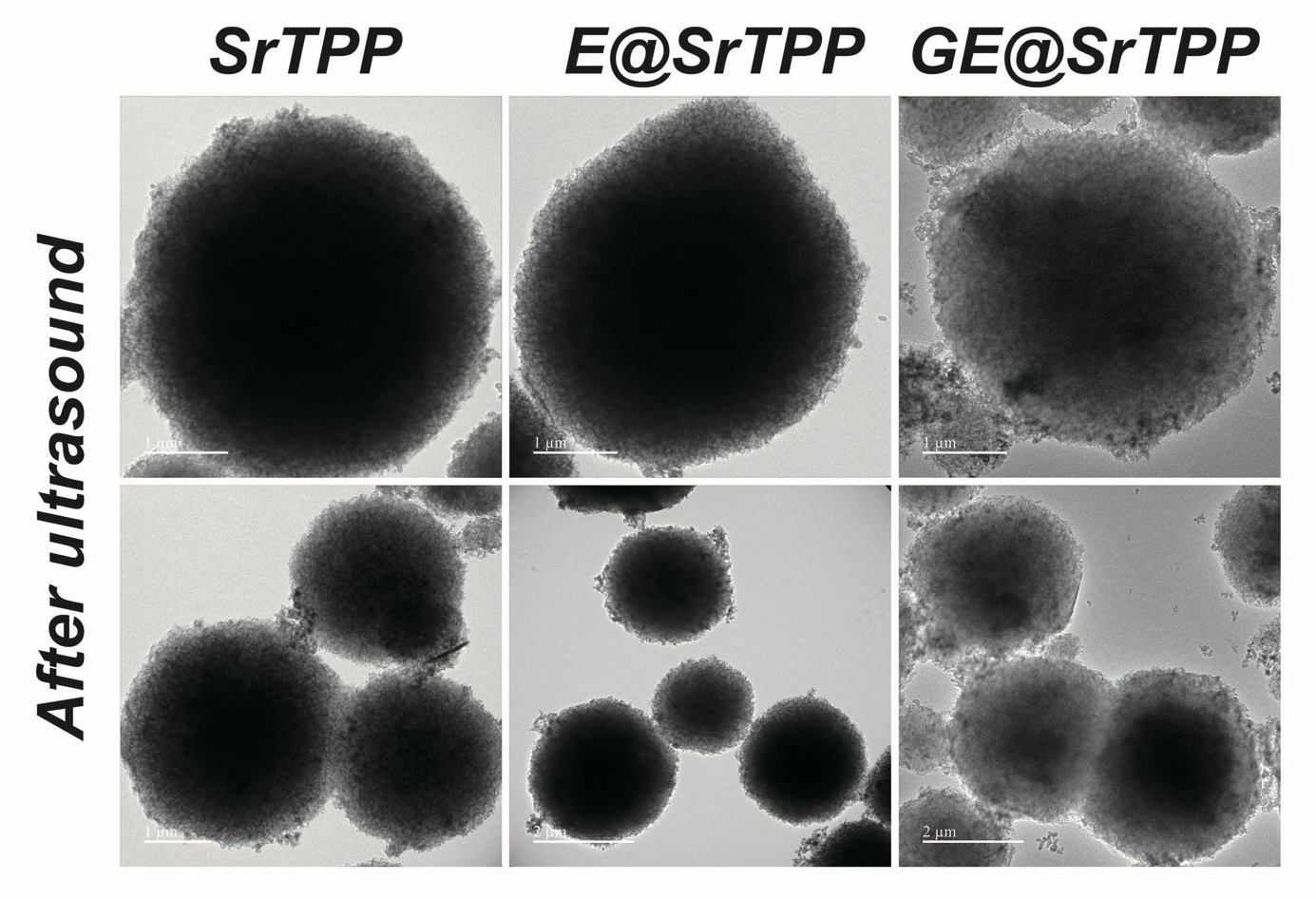


**Figure S9**. TEM images of each group of materials after ultrasound treatment.


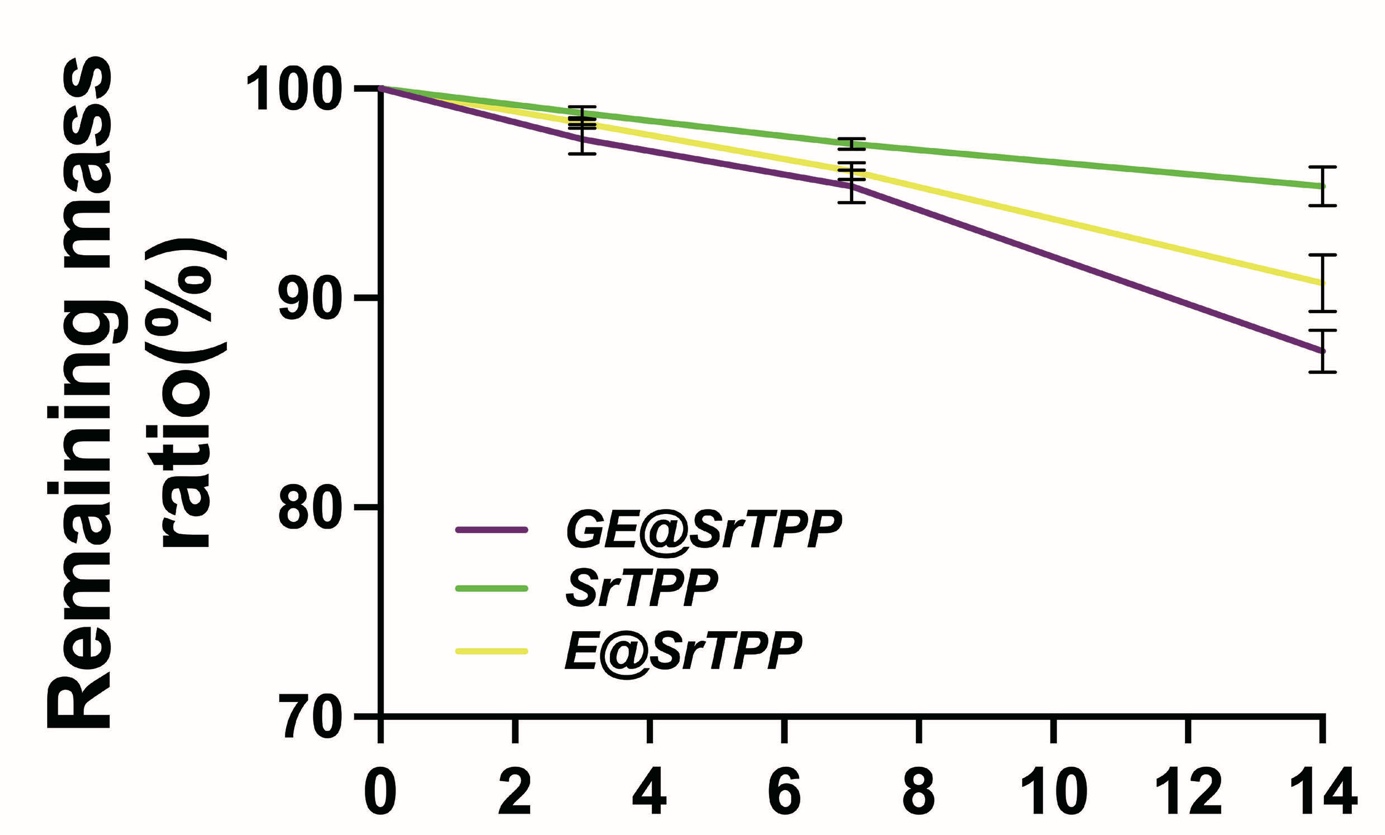


**Figure S10**. Degradation rates of each group of materials within 14 days.


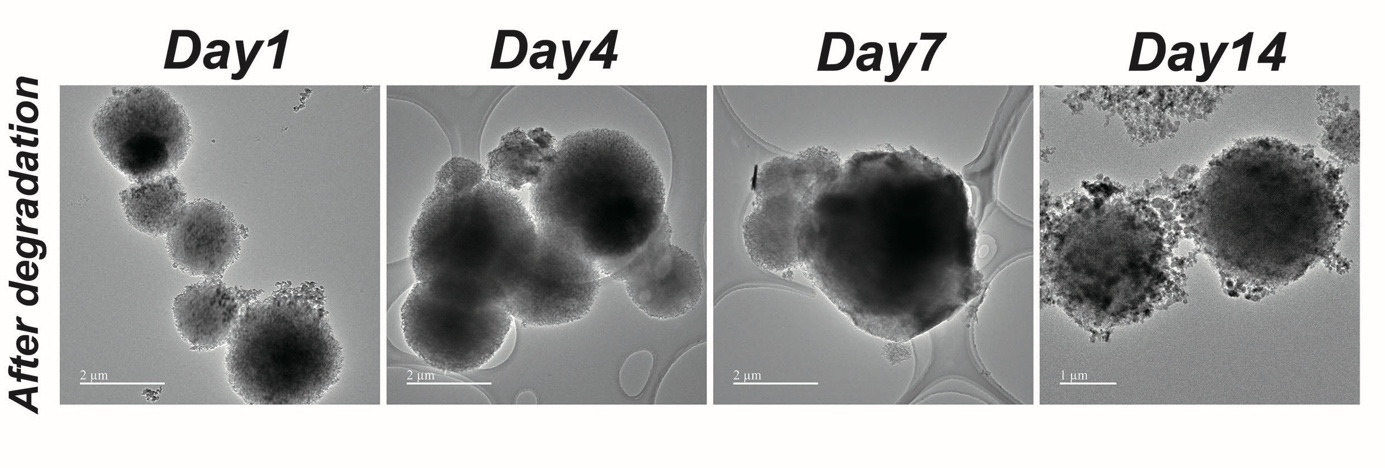


**Figure S11**. The morphology of GE@SrTPP during degradation for day 1, day 3, day 7 and day 14.


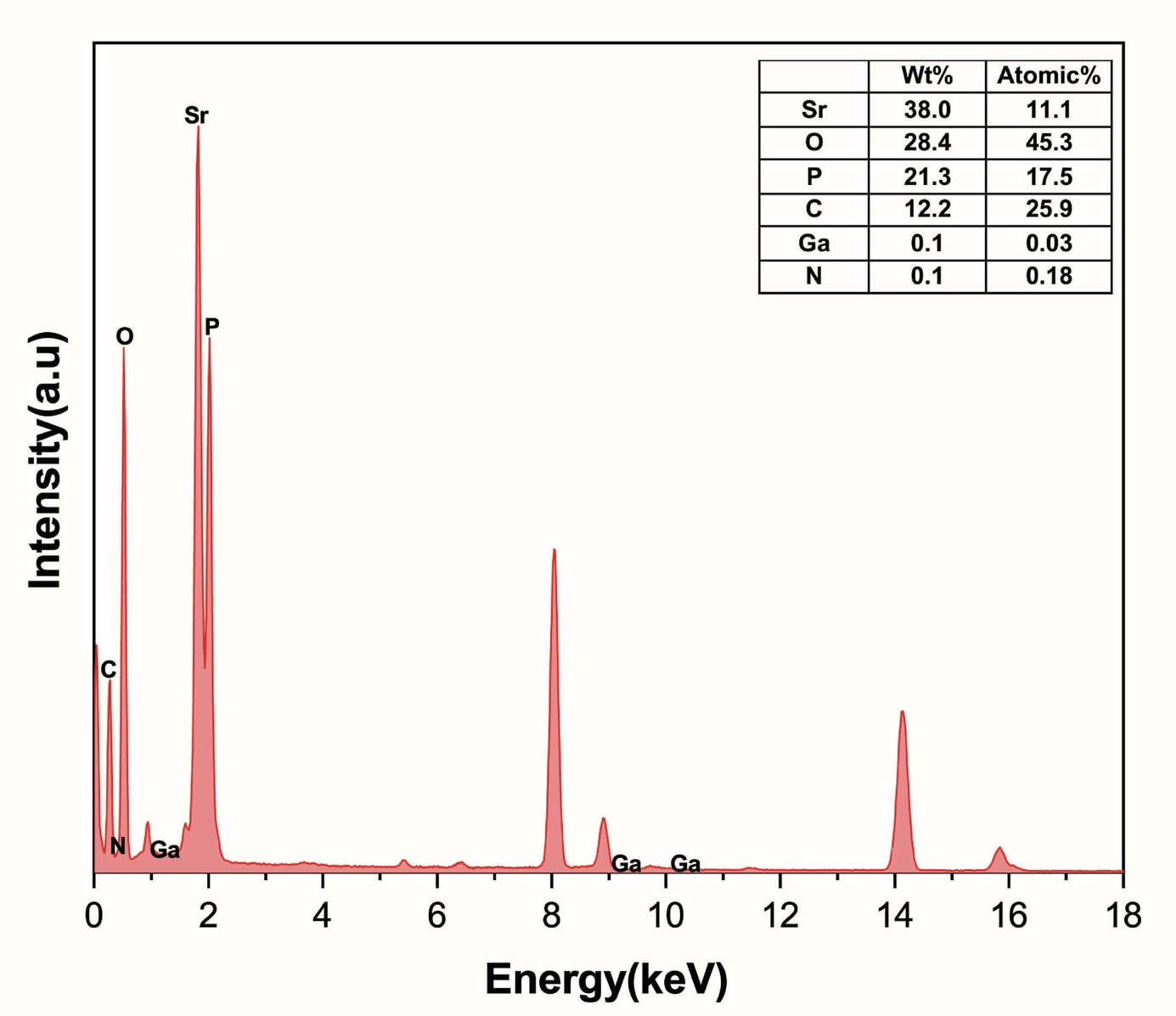


**Figure S12**. EDS analysis of GE@SrTPP after 14 days of degradation.


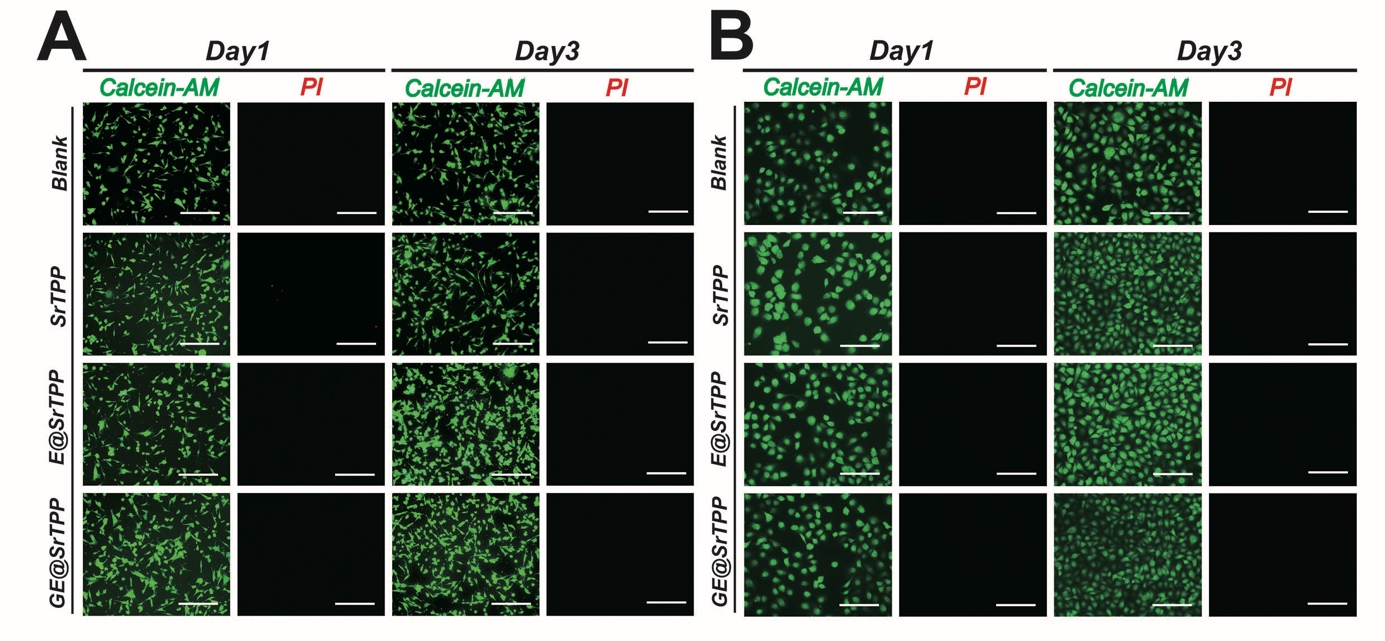


**Figure S13.** (A) Live/dead staining analysis of the effect of the 1/16 dilution ratio extract on the cell viability of L929 cells; (B) The live/dead staining analysis of the effects of the 1/16 dilution ratio extract on the cell viability of HUVECs cells.

**
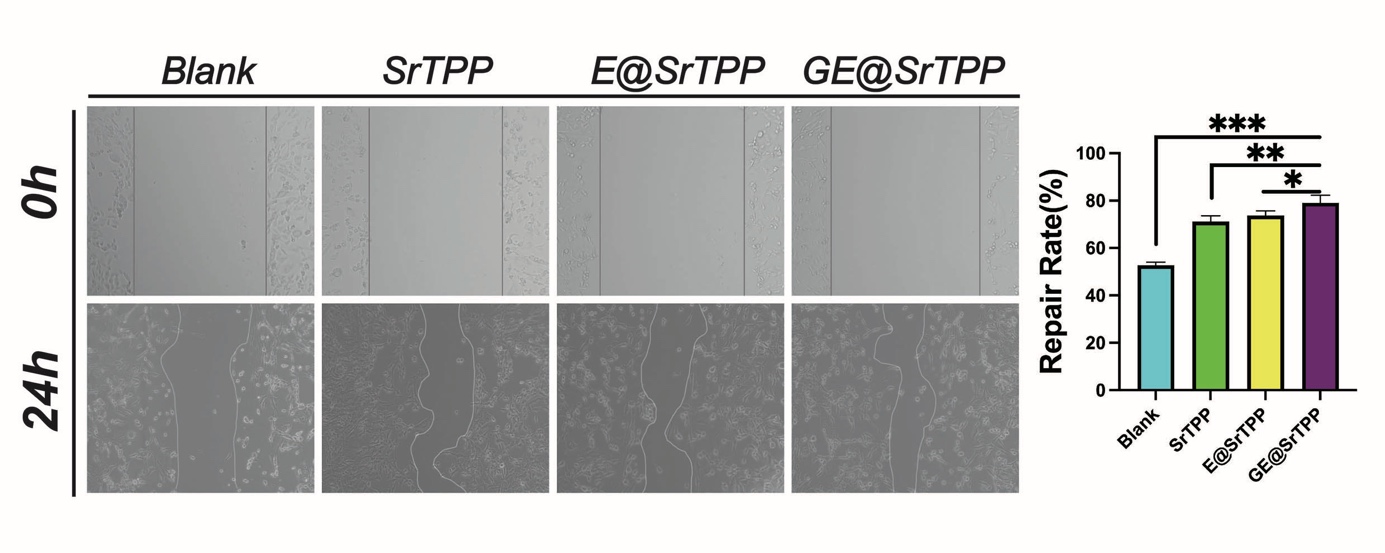
****Figure S14**. The effect of various groups of microparticles on L929 cell migration.


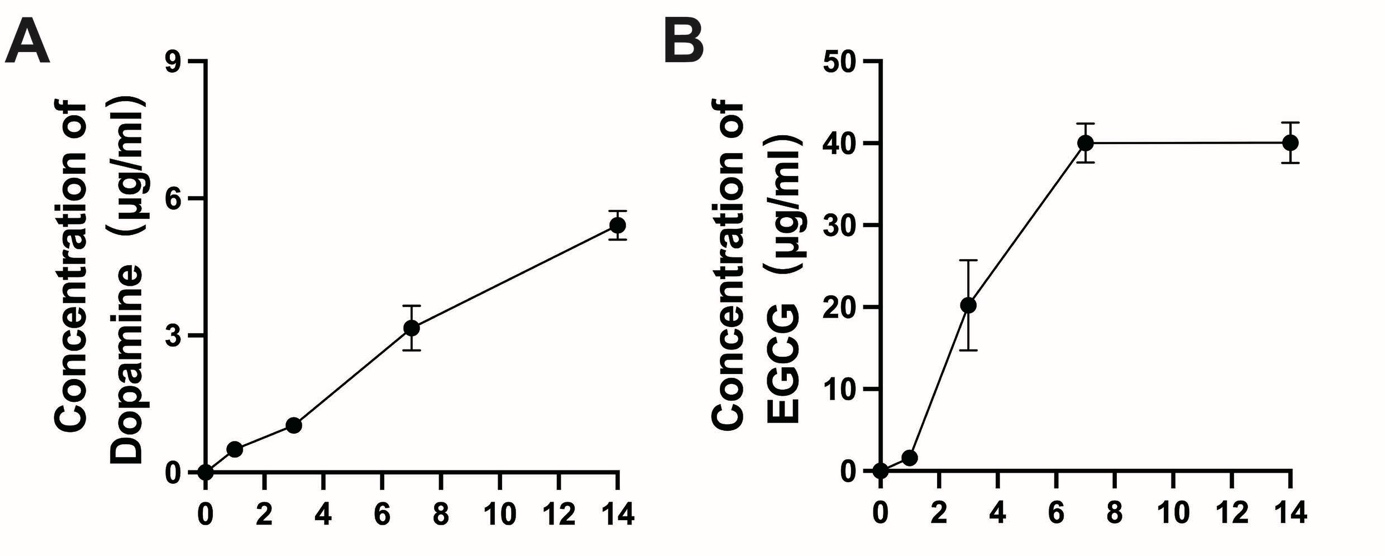


**Figure S15.** The release curves of EGCG and Dopamine.

**
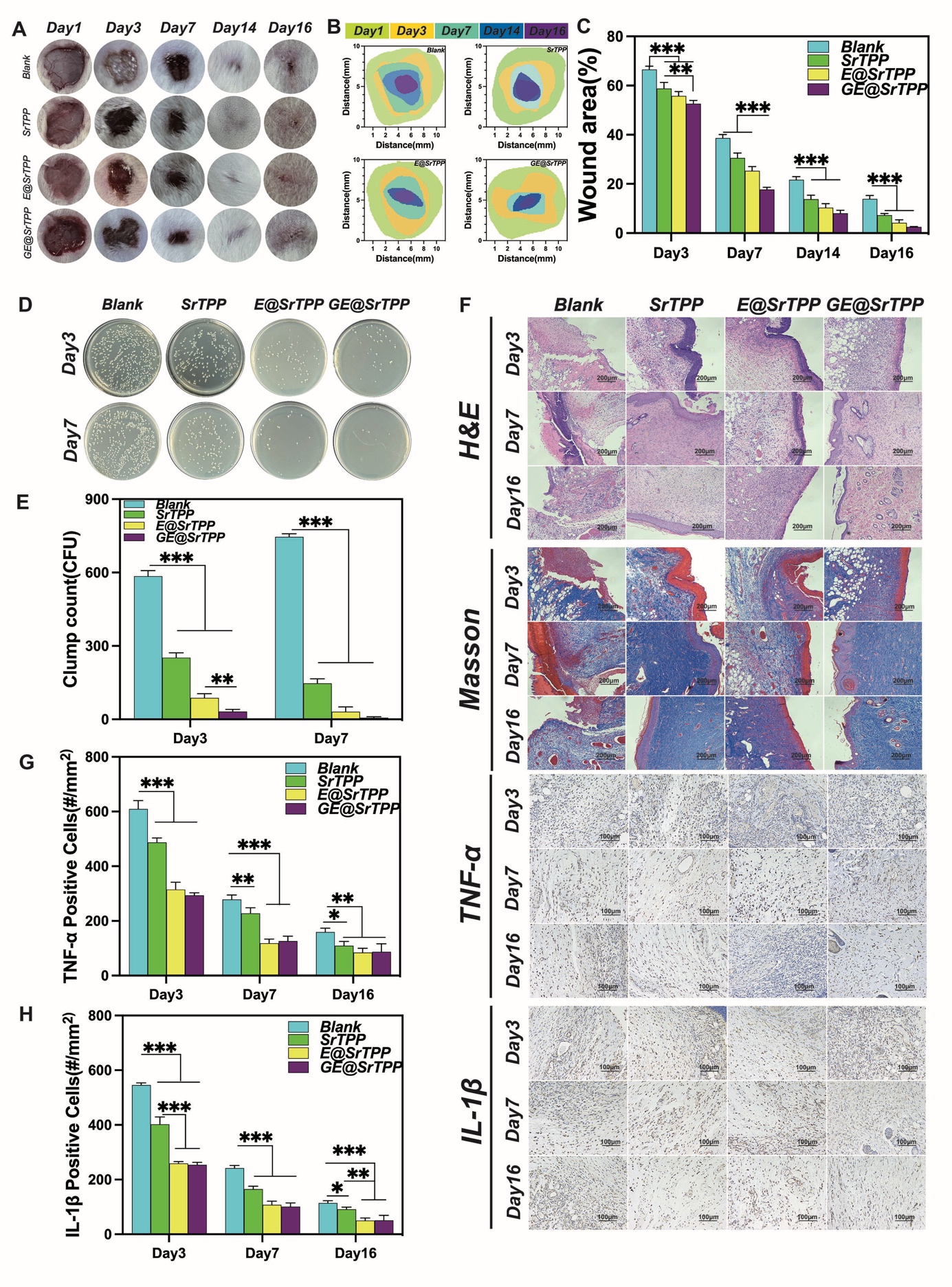
****Figure S16**. GE@SrTPP promotes the repair and regeneration of chronic full-layer wound caused by bacterial infection *in vivo*. (A) Representative images and diagrams of the reaction of the particles on days 1, 3, 7, 14 and 16. (B) Schematic images of wound healing area. (C) Quantitative analysis of wound healing rate in each group. (D) Images of bacteria coating and (E) relevant quantitative analysis on days 3 and 7. (F) H&E and Masson staining were performed on day 3, 7 and 16 of each group. (G) Quantitative analysis of TNF-α positive cells and (H) IL-1β positive cells (n = 3, * P < 0.05; ** p < 0.01; *** p < 0.001).


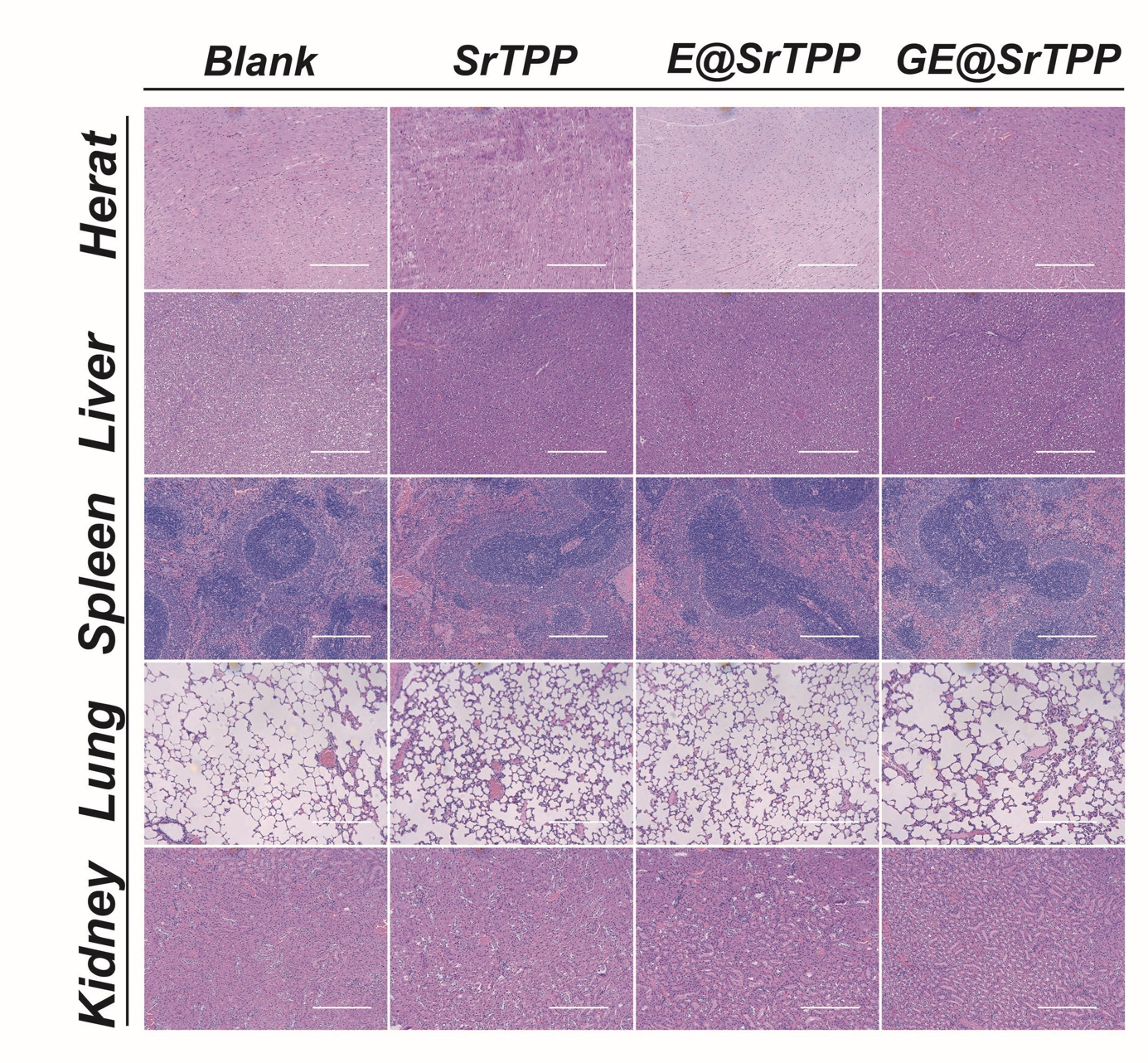


**Figure S17**. 14 days after infected skin wound repair, HE staining analysis showed the effect of GE@SrTPP on various organs of animal models. (bar=200μm)

**
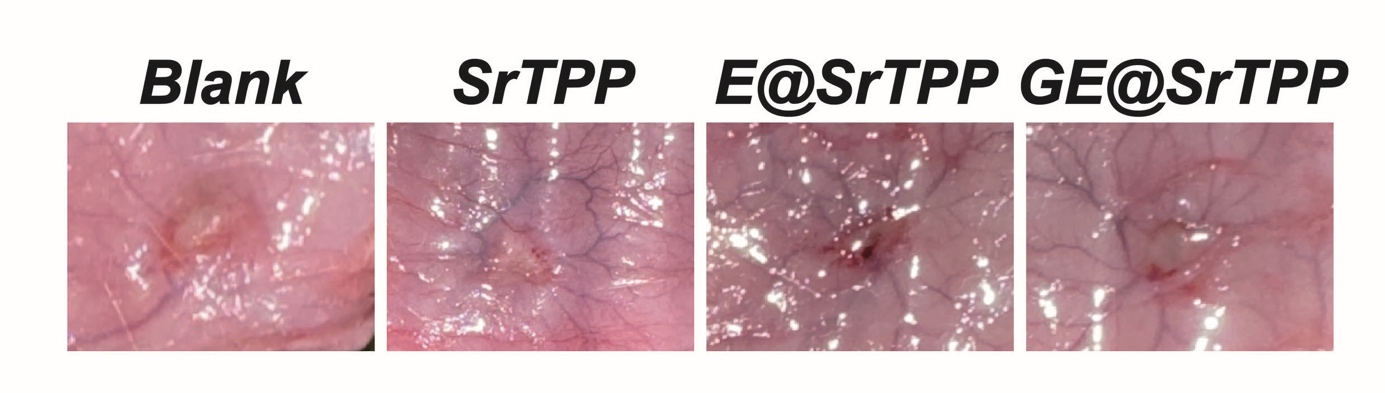
**

**Figure S18**. Gross view of wound neovascularization in different treatment groups.

**
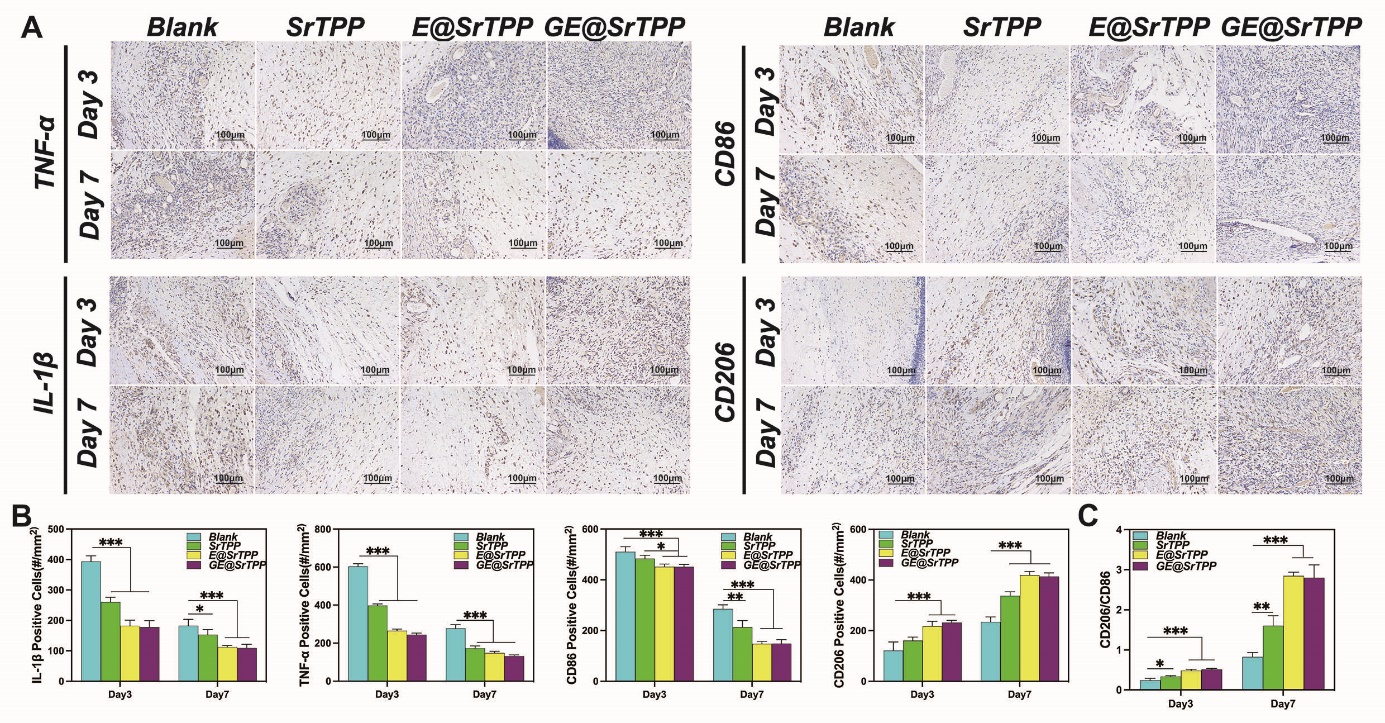
** **Figure S19**．(A) Immunohistochemistry for TNF-α, IL-1β, CD86, and CD206 at 3 and 7 days. (B) TNF-α, IL-1β, CD86 and CD206 positive cells. (C) Ratio of M2 polarized macrophages to M1 polarized macrophages. (n = 3, * P < 0.05; ** p < 0.01; *** p < 0.001).


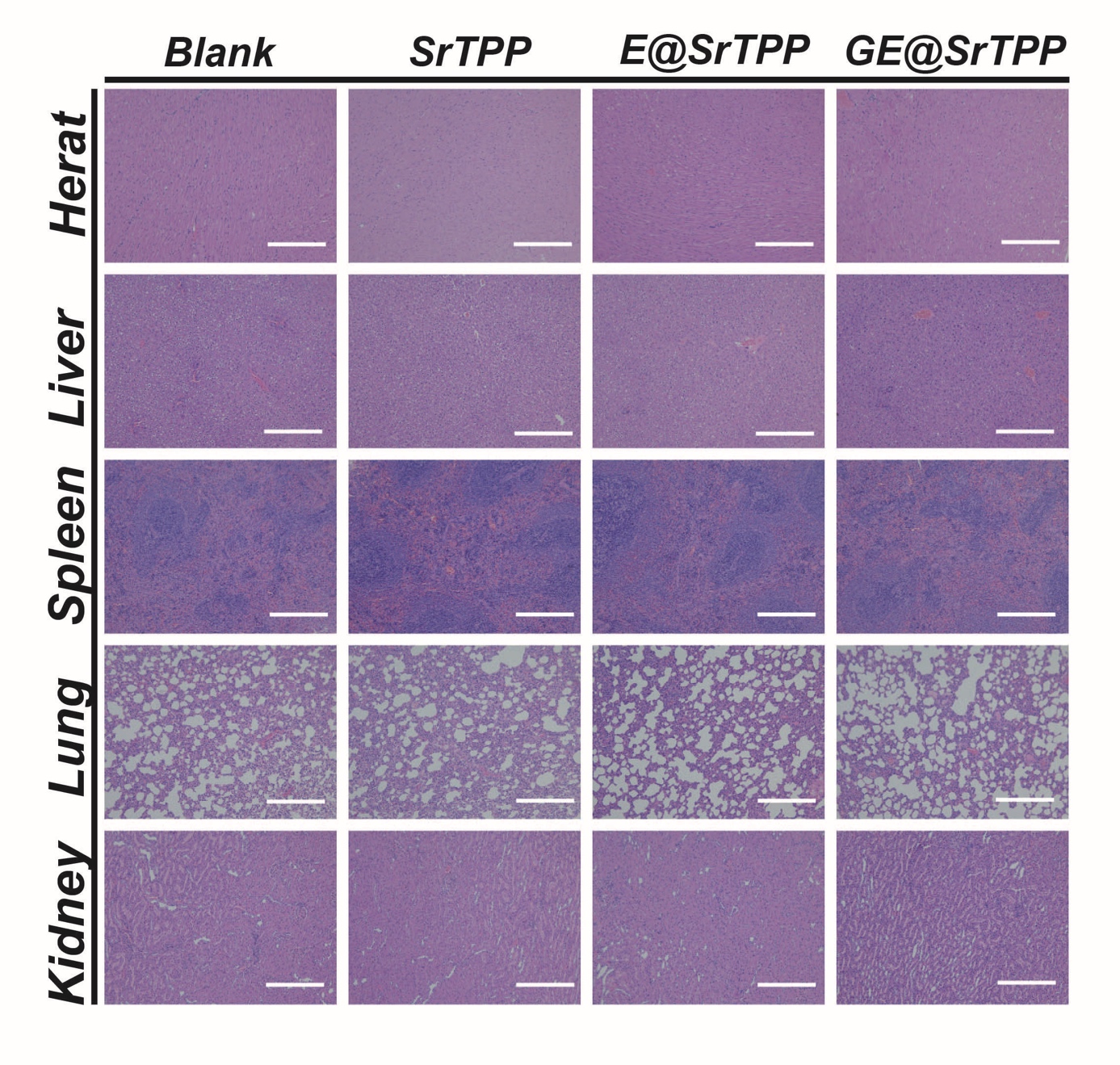


**Figure S20.** 14 days after skin repair, HE staining analysis showed the effect of GE@SrTPP on various organs of animal models. (bar=200μm)


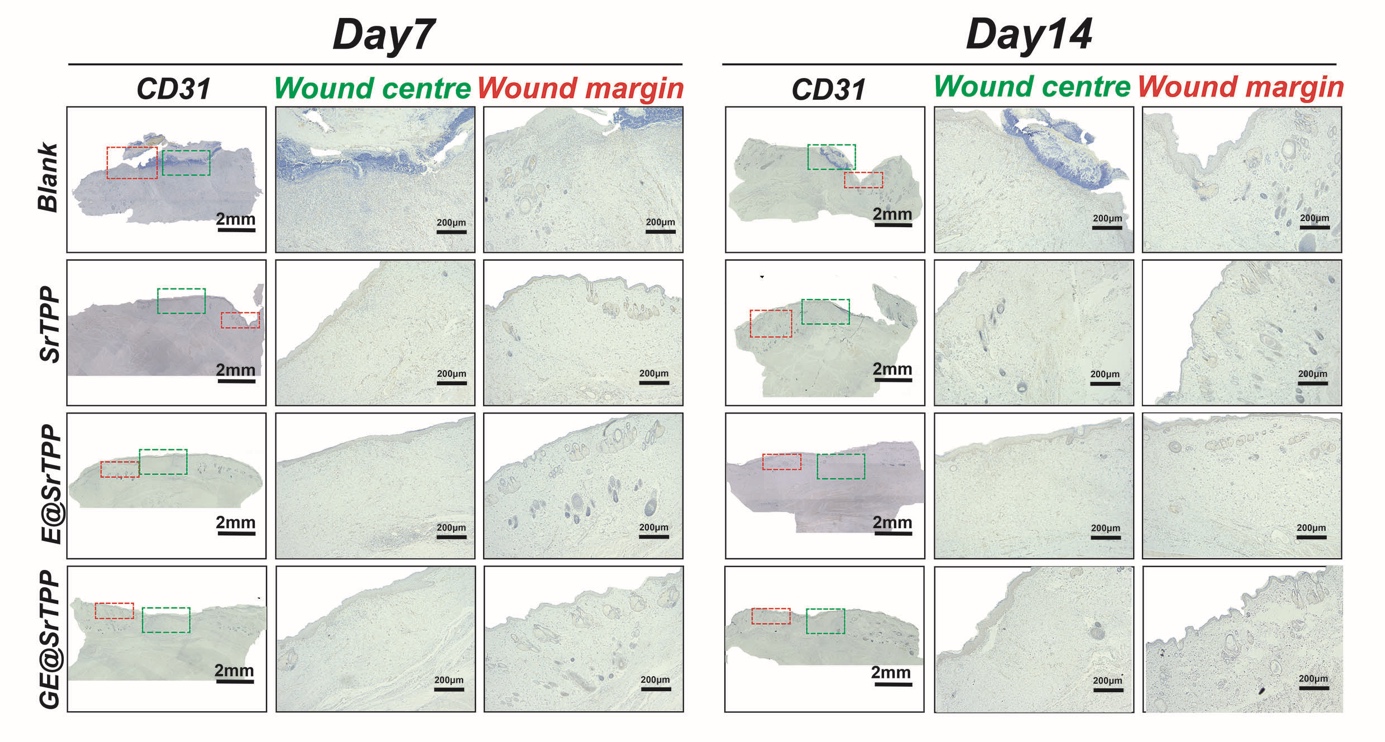
**Figure S21.** Immunohistochemical (IHC) analysis of the CD31 level in the repaired wound.


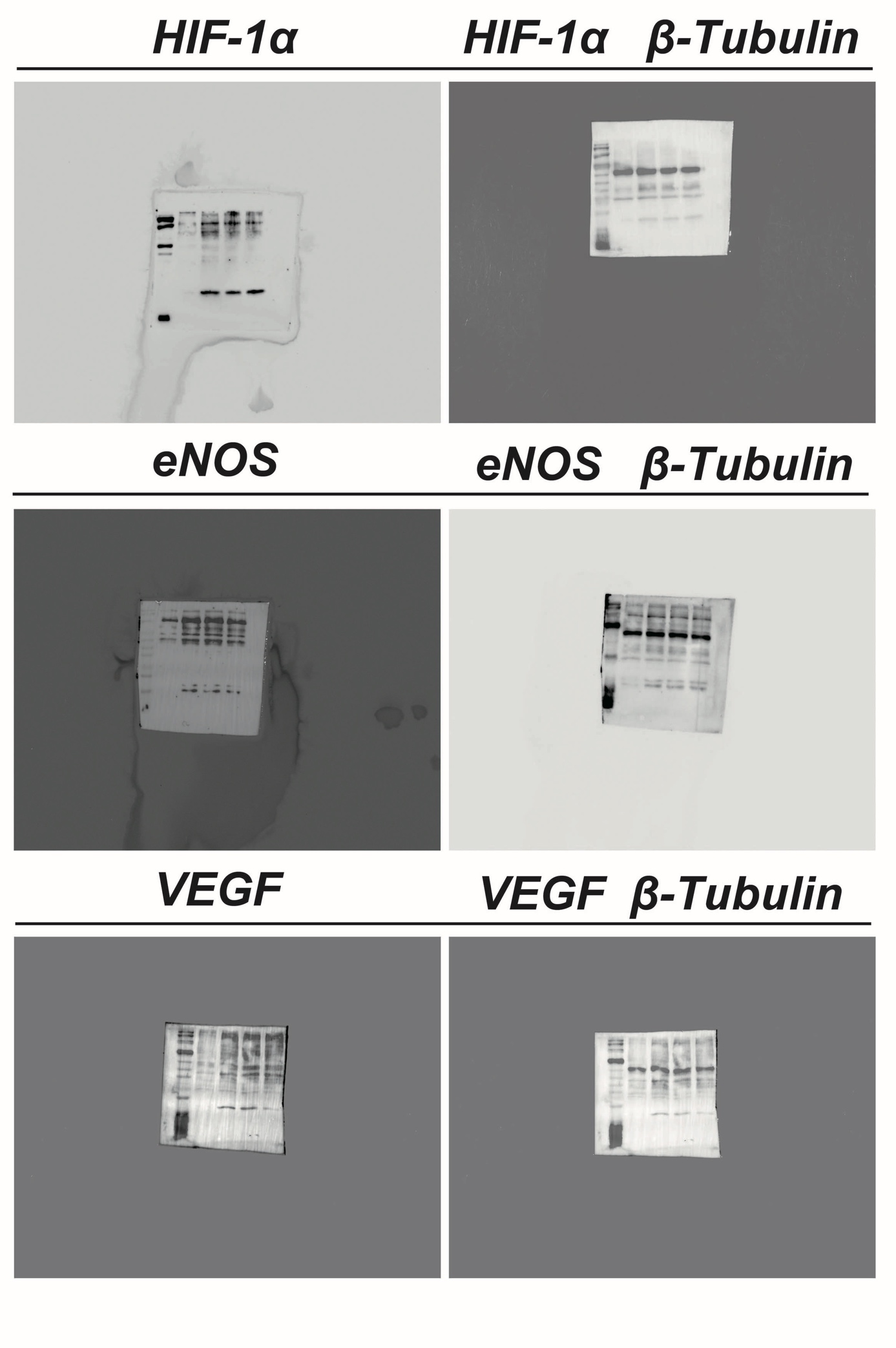


**Figure S22.** The Raw data for WB (Fig. 6I).

**Table S1.** The primer sequences used in the Q-PCR of this study.

| Raw 264.7 | | |
| --- | --- | --- |
| Gene |  | Primer sequence |
| CCR7 | Forward | CCATGACGGATACCTACCTGCT |
|  | Reverse | CCCTTACACAGGTAGACGCCAA |
| CD206 | Forward | ATCCACGAGCAAATGTACCTCA |
|  | Reverse | TAGCCAGTTCAGATACCGGAA |
| iL-1β | Forward | CTACCTGTGTCTTTCCCGTG |
|  | Reverse | TTTGTTGTTCATCTCGGAGC |
| Arg-1 | Forward | ATCAACACTCCCCTGACAACC |
|  | Reverse | TCGCAAGCCAATGTACACGAT |
| iNOS | Forward | ACGCTTCACTTCCAATGCAAC |
|  | Reverse | CAGCCTCATGGTAAACACGTTC |
| iL-10 | Forward | GAGAAGCATGGCCCAGAAATC |
|  | Reverse | GAGAAATCGATGACAGCGCC |
| iL-1ra | Forward | AGAGCCCCTTATAGTCACGAA |
|  | Reverse | TACACCCTGCAAAAGTTGTTCC |
| TNF-α | Forward | CTGTAGCCCACGTCGTAGCAA |
|  | Reverse | TGTCTTTGAGATCCATGCCGTT |
| iL-6 | Forward | TCCAGTTGCCTTCTTGGGAC |
|  | Reverse | GTACTCCAGAAGACCAGAGG |
| GAPDH | Forward | AGAACATCATCCCTGCATCCAC |
|  | Reverse | TCAGATCCACGACGGACACA |

**Table S2.** Concentrations of Sr²⁺ and Ga³⁺ released from GE@SrTPP over various days, as determined by ICP-MS.

|  | Sr(mg/L) | Ga(mg/L) |
| --- | --- | --- |
| Day1 | 3.7 | 5.2 |
| Day3 | 14.7 | 11.8 |
| Day7 | 24.6 | 13.4 |
| Day14 | 38.7 | 14.8 |

**Table S3.** The antibacterial rate of the previous reported materials.

| Materials | Antibacterial rate | | References |
| --- | --- | --- | --- |
|  | S.aureus | E.coli |  |
| HA-ADP/UCS | 99.55% | 99.97% | [75] |
| CPG-B3 | 98.99±1.16% | 59.40± 4.10% | [52] |
| GCQCNF-5 | 99.998% | 99.998% | [63] |
| GA/OKGM/MT | 91.2% | 89.9% | [73] |
| CT/Ag-MNs | 99.99% | 99.99% | [94] |
| BSP-U/DAHA-1 | 94.94 ± 0.52% | 91.68 ± 1.88% | [95] |
| CSB@TA | 99.5% | 98.6% | [96] |
| γ-PGA | 70% | 68% | [97] |

**Table S4**. Hemostatic data from previous reported hemostatic materials.

|  | Materials | Hemostasis time(s) | Blood loss(mg) | References |
| --- | --- | --- | --- | --- |
| Rat liver | H(C-A/OD/TA/HNT2) | 55.7 ± 8.6 | 64.6 ± 16.2 | [71] |
|  | QCSMA/DAMA3/Zn-nWH3 | 129 ± 22 | 27 ± 5 | [53] |
|  | SA/CNF/PDA | 95 | 19.73 | [49] |
|  | CPN31 | 87 ± 6.2 | 87±3 | [62] |
|  | CPG-B3 | 10.7 ± 1.2 | 90 ± 12 | [52] |
|  | PCT-5 | 23.0 ± 2.6 | 83.0 ± 19.1 | [72] |
|  | GA/OKGM/MT | 85.3 | 146.5 | [73] |
|  | GCQCNF-5 | 52.5 ± 3.7 | 58.4 ± 1.8 | [63] |
|  | P10P8TY0.1 | 144.5 ± 40.6 | 26.3 ± 8.6 | [64] |
| Rat rail | GCQCNF-5 | 67.7 ± 2.2 | 70.7 ± 3.6 | [63] |
|  | CA/SP@Th | 72.67 | 55 | [74] |
|  | CMCS/SA-K60 | 96.66 ± 5.77 | 27.66 ± 3.05 | [65] |
|  | HA-ADP/UCS | 46 | 35 | [75] |
|  | 2CS-DAC | 163.3±5.8 | 49.6±16.0 | [66] |
|  | CA/SP@AgNs | 109 | 115 | [67] |
|  | TSD10 | 160 ± 72 | 60 ± 30 | [68] |
|  | PSLMs | 81.20 | 43.33 | [69] |

**Table S5.** Wound closure and remodeling of other materials (previous reported hemostatic materials) at 14 days.

| Materials | Wound closure | epidermal thickness(μm) | References |
| --- | --- | --- | --- |
| H(P+T) | ~88% | 90 | [98] |
| KBP@KH | ~92% | ~40 | [39] |
| NG/CMCS/HA/SF | ~98% | ~170 | [99] |
| TPP | ~92% | ~120 | [100] |
| P-LP-PMX-CA-L@E | ~90% | ~30 | [101] |
| BG1O@PG | ~97% | 16 | [102] |
| G4/Aloin-KGM | ~92% | ~30 | [103] |
| 4% OGLP-CMC/SA | 90% | 64 | [104] |

**Reference**

94. Yang X, Jia M, Li Z, Ma Z, Lv J, Jia D, He D, Zeng R, Luo G, Yu Y. In-situ synthesis silver nanoparticles in chitosan/Bletilla striata polysaccharide composited microneedles for infected and susceptible wound healing. *International Journal of Biological Macromolecules* 2022;215:550-559.

95. Yue X, Zhao S, Qiu M, Zhang J, Zhong G, Huang C, Li X, Zhang C, Qu Y. Physical dual-network photothermal antibacterial multifunctional hydrogel adhesive for wound healing of drug-resistant bacterial infections synthesized from natural polysaccharides. *Carbohydrate Polymers* 2023;312.

96. Wang N, Tian X, Cheng B, Guang S, Xu H. Calcium alginate/silk fibroin peptide/Bletilla striata polysaccharide blended microspheres loaded with tannic acid for rapid wound healing. *International Journal of Biological Macromolecules* 2022;220:1329-1344.

97. Zhu L, Chen J, Mao X, Tang S. A γ-PGA/KGM-based injectable hydrogel as immunoactive and antibacterial wound dressing for skin wound repair. *Materials Science and Engineering: C* 2021;129.

98. Zou C-Y, Lei X-X, Hu J-J, Jiang Y-L, Li Q-J, Song Y-T, Zhang Q-Y, Li-Ling J, Xie H-Q. Multi-crosslinking hydrogels with robust bio-adhesion and pro-coagulant activity for first-aid hemostasis and infected wound healing. *Bioactive Materials* 2022;16:388-402.

99. Yang H, Xu H, Lv D, Li S, Rong Y, Wang Z, Wang P, Cao X, Li X, Xu Z, Tang B, Zhu J, Hu Z. The naringin/carboxymethyl chitosan/sodium hyaluronate/silk fibroin scaffold facilitates the healing of diabetic wounds by restoring the ROS-related dysfunction of vascularization and macrophage polarization. *International Journal of Biological Macromolecules* 2024;260.

100. Zhao H, Lou Z, Chen Y, Cheng J, Wu Y, Li B, He P, Tu Y, Liu J. Tea polyphenols (TPP) as a promising wound healing agent: TPP exerts multiple and distinct mechanisms at different phases of wound healing in a mouse model. *Biomedicine & Pharmacotherapy* 2023;166.

101. Jing Y, Huang T, Zhao B, Zhao L, Zhang N, Zhang K, Wang K, Wang J, Hua J, Tu Q. A ROS/glucose stimulated-responsive ADSCs-derived exosomes-release hydrogel system for diabetic wound healing. *Chemical Engineering Journal* 2024;487.

102. Yuan Z, Zhang L, Jiang S, Shafiq M, Cai Y, Chen Y, Song J, Yu X, Ijima H, Xu Y, Mo X. Anti-inflammatory, antibacterial, and antioxidative bioactive glass-based nanofibrous dressing enables scarless wound healing. *Smart Materials in Medicine* 2023;4:407-426.

103. Zhang W, Chen H, Zhao J, Chai P, Ma G, Shi X, Dong Y, Jiang Y, Zhang Q, Hu Z, Wei Q. A guanosine/konjac glucomannan supramolecular hydrogel with antioxidant, antibacterial and immunoregulatory properties for cutaneous wound treatment. *Carbohydrate Polymers* 2024;326.

104. Li F, Liu T, Liu X, Han C, Li L, Zhang Q, Sui X. Ganoderma lucidum polysaccharide hydrogel accelerates diabetic wound healing by regulating macrophage polarization. *International Journal of Biological Macromolecules* 2024;260.
